# Matrix Isolation Study of Fumaric and Maleic Acids in Solid Nitrogen

**DOI:** 10.1021/acs.jpca.2c02770

**Published:** 2022-06-23

**Authors:** Timur Nikitin, Susy Lopes, Rui Fausto

**Affiliations:** † CQC-IMS, Department of Chemistry, University of Coimbra, 3004-535 Coimbra, Portugal

## Abstract

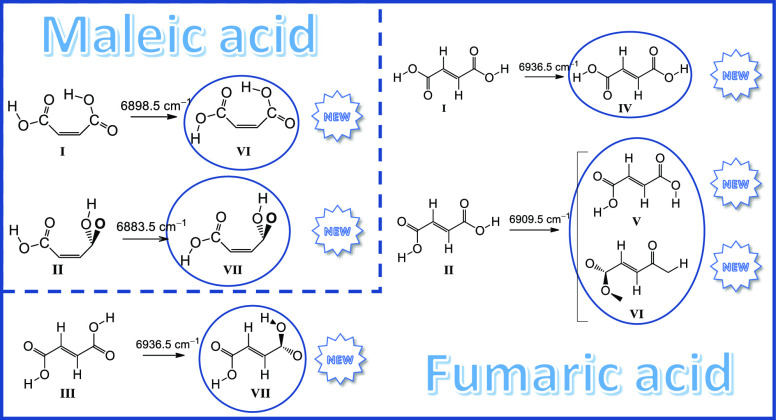

Fumaric and maleic acids ((*E*)- and (*Z*)-HOOC–CH=CH–COOH, FA and MA) were studied experimentally by infrared spectroscopy in nitrogen matrixes and theoretically by quantum chemical calculations. The calculations, carried out at the DFT(B3LYP) and MP2 levels of theory, predicted the existence of at least 5 conformers of maleic acid and 10 conformers of fumaric acid. After the deposition of the matrixes, two conformers of maleic acid (**I** and **II**) and three conformers (**I**–**III**) of fumaric acid were observed and characterized vibrationally. Selective narrowband near-infrared (NIR) excitation of the first OH stretching overtones of the different conformers of maleic and fumaric acids initially present in the matrixes allowed the generation of higher-energy forms, never before observed experimentally. In the case of maleic acid, conformers **I** (a *cis–trans* form, where *cis* and *trans* designate the conformation of the carboxylic groups of the molecule) and **II** (*cis–cis*) were found to generate the novel conformers **VI** (*trans–trans*) and **VII** (*cis–trans*), respectively. The conversion of conformer **II** into the most stable conformer **I** was also observed. For fumaric acid, the *cis–cis* conformers **I**–**III** were found to give rise to the new *cis–trans* conformers **IV**–**VII**, respectively. The tunneling decay of the new conformers produced upon NIR excitation of the lowest-energy conformers initially trapped in the matrixes was observed, and their lifetimes in solid N_2_ were determined. The increased stability of all of the observed high-energy conformers of the studied acids in the N_2_ matrix, compared to the argon matrix, where they could not be observed experimentally, demonstrates the stabilizing effect of the interaction between the OH groups of the acids with the matrix N_2_ molecules, in line with previous observations for other carboxylic acids. In addition, the photochemistry of matrix-isolated maleic and fumaric acids upon broad-band UV irradiation (λ > 235 nm) was also investigated. UV-induced isomerization of both acids around the C=C double bond was observed, together with their decarboxylation to acrylic acid.

## 1 Introduction

Fumaric acid (FA, (*E*)-HOOC–CH=CH–COOH, also known as *trans*-butenedioic acid) and maleic acid (MA, (*Z*)-HOOC–CH=CH–COOH, or *cis*-butenedioic acid) are the simplest dicarboxylic acids bearing a C=C bond. Both FA and MA are naturally occurring substances^1,2^ that receive applications in different areas, e.g., as corrosion inhibitors,^3^ feedstock in the production of polyester resins,^4−6^ and antiscaling agents.^7,8^ They are also used in the food and cosmetics industries^9−11^ and in medicine.^12,13^ However, the use of maleic acid as a food additive has been banned in both Europe and the USA because of its potential toxicity to the kidneys.^14^

Considering their practical relevance, the two compounds have been the subject of a considerable number of studies. Their crystalline structures have been reported long ago,^15−20^ and the molecular structure of FA has been studied by both gas-phase electron diffraction and microwave spectroscopy.^21−23^ In addition, the infrared (IR) and ultraviolet (UV) spectra of MA and FA in aqueous solution have been described,^24,25^ as well as their room-temperature Raman spectra.^26^ The mechanism of thermal isomerization of MA into FA is a classic classroom example of a *cis*–*trans* isomerization and has been investigated early on.^27−31^

The first low-temperature matrix isolation study of monomeric maleic and fumaric acids was undertaken back in 2001, in solid argon, in a study where the experimental data have been interpreted with help of *ab initio* and density functional theory (DFT) calculations.^32,33^ In that work, Maçôas et al.^32^ reported on the existence of a total of five putative conformers for each compound, as predicted computationally. The most stable conformer of maleic acid (according to the reported MP2 and DFT calculations)^32^ was shown to exhibit a nearly planar structure, with one of the carboxylic groups in the *cis* conformation (O=C–O–H dihedral angle equal to 0°) and the other in the *trans* conformation (O=C–O–H dihedral angle equal to 180°), with the latter being hydrogen bonded to the first through a strong O–H···O= intramolecular H-bond.^32^ On the other hand, the second most stable conformer of maleic acid was shown to have both carboxylic groups in the *cis* arrangement, one in the main molecular plane and the second approximately perpendicular to that plane.^32^ These two conformers were observed experimentally in the argon matrix, with the less stable conformer converting to the most stable form upon narrowband infrared irradiation at 6901 cm^–1^ (first overtone of the νOH stretching mode of the less-stable conformer).^32^ In the case of fumaric acid, three low-energy conformers exhibiting both carboxylic groups in the *cis* arrangement were found computationally and were experimentally observed. It is noteworthy that the attempts to promote conformational conversions between the conformers of fumaric acid isolated in solid argon via IR excitation were unsuccessful.^32,33^

In a subsequent publication, the same authors analyzed the photoinduced rotamerization in several dicarboxylic acids in solid argon, including fumaric and maleic acids, and concluded that rotamerization around the C–C bonds was observed only when the precursor forms subjected to the IR pumping have a nonplanar heavy atom skeleton, possibly due to the effect of a more restrictive packing of the matrix lattice atoms around the planar conformers that hinders the internal rotation when it involves an extensive motion of heavy atoms.^34^

The molecular structure of fumaric acid was recently reinvestigated theoretically by Vogt et al.^22^ MP2/cc/pVTZ calculations predicted six conformers for the compound, with three of them being *cis*–*cis* forms with relative energies within *ca*. 3 kJ mol^–1^. The geometries of these low-energy conformers were reinvestigated at a higher level of calculation (coupled cluster with single, double, and triple excitations; CCSD(T)) in a subsequent study.^23^

In the present investigation, the IR spectra of monomeric FA and MA isolated in low-temperature (15 K) nitrogen matrixes were obtained. The spectroscopic and structural information obtained on these acids in the present investigation is enriched compared to the previous studies performed in solid argon.^32,33^ In particular, *in situ* selective narrowband near-IR (NIR) excitation of the conformers of both MA and FA initially present in the matrixes allowed us to successfully generate new conformers that have never been reported experimentally hitherto. It shall be highlighted that conformational conversions were observed in this study for both fumaric and maleic acids, as opposed to what has been previously reported for the compounds in solid argon, where only maleic acid was observed to undergo conformational changes. Tunneling decays of the newly generated conformers and the effects of *in situ* broad-band UV irradiation (λ > 235 nm) of the matrix-isolated compounds were also investigated for both acids. The experimental results were interpreted with the help of theoretically obtained data resulting from quantum chemical calculations performed within the DFT(B3LYP) and MP2 theoretical frameworks, with the 6-311++G(d,p) and 6-311++G(2d,2p) basis sets, which also allowed the undertaking of a more complete conformational study of monomeric FA and MA than those previously reported.^22,23,32,33^

## 2 Computational Details

The quantum chemical calculations were performed with the Gaussian 09 (revision A.02)^35^ and Gaussian 16 (revision B.01)^36^ program packages at the DFT(B3LYP)^37−39^ and MP2^40^ levels of theory using the 6-311++G(d,p) and 6-311++G(2d,2p) basis sets.^41−43^ All geometries were optimized using the TIGHT convergence criteria, and the nature of all described stationary points was further characterized through the analysis of the corresponding Hessian matrices. The optimized structures of all conformers described in this study were confirmed to correspond to true minimum-energy conformations on the different potential energy surfaces investigated.

Two-dimensional maps describing the conformational landscapes of the studied molecules were calculated by performing relaxed scans on the corresponding potential energy surfaces along the relevant coordinates (2D scans), and the transition-state structures and energies for the relevant conformational interconversions were obtained using the synchronous transit-guided quasi-Newton method.^44^

Anharmonic IR spectra were computed using the fully automated second-order vibrational perturbative approach of Barone and co-workers, allowing for the evaluation of anharmonic infrared intensities of up to 2 quanta, including overtones and combination tones.^45−48^ The resulting wavenumbers, together with the calculated IR intensities, were used to simulate the spectra shown in the figures, through convolution with Lorentzian functions having a full width at half-maximum (fwhm) equal to 2 cm^–1^. The vibrational analysis was supported by the animation of the vibrations of all calculated species provided by the Chemcraft software.^49^

## 3 Experimental Details

Maleic and fumaric acids were obtained from Aldrich (purity >99%). The matrixes were prepared by codeposition of the sublimated compounds together with a large excess of the matrix host gas [N_2_ (N60) obtained from Air Liquide] onto a CsI substrate placed at the cold (15 K) tip of the cryostat (an APD Cryogenics closed-cycle helium refrigeration system, with a DE-202A expander, or an ARS Cryogenics closed-cycle helium refrigeration system, with a DE-202SI expander). The studied acids were placed in a specially designed homemade temperature-variable minioven attached to the cryostat through a needle valve. The temperature used to sublimate the compounds (ca. 325–330 K) was monitored by a thermocouple integrated inside the oven, while the valve nozzle was kept at room temperature (298 K). The temperature of the CsI window was measured directly at the sample holder by a silicon diode sensor connected to a digital temperature controller (Scientific Instruments, model 9650-1 and LakeShore 335), which provides an accuracy of 0.1 K.

The IR spectra were obtained in the 4000–400 cm^–1^ range, with 0.5 cm^–1^ spectral resolution, using either a Thermo Nicolet 6700 FTIR spectrometer equipped with a deuterated triglycine sulfate (DTGS) detector and a Ge/KBr beam splitter or a Thermo Nicolet iS50 FTIR spectrometer (UV experiments) equipped with an MCT/A detector and a KBr beam splitter. To avoid interference from atmospheric H_2_O and CO_2_, a stream of dry CO_2_-filtered air was continuously purging the optical path of the spectrometers.

The matrixes were irradiated in the NIR region with a Quanta-Ray MOPO-SL optical parametric oscillator pumped with a pulsed Nd:YAG laser (pulse energy 10 mJ, duration 10 ns, and repetition rate 10 Hz). Broadband UV irradiation of the matrixes was carried out with UV light provided by a 500 W high-pressure Hg(Xe) lamp (Newport, Oriel Instruments), with output power set to 250 W, through the outer KBr window of the cryostat and a water filter (λ > 235 nm, as defined by the onset of KBr transmission in the UV).

## 4 Results and Discussion

### 4.1 Geometries and Energies

#### 4.1.1 Maleic Acid

**Figure 1:**
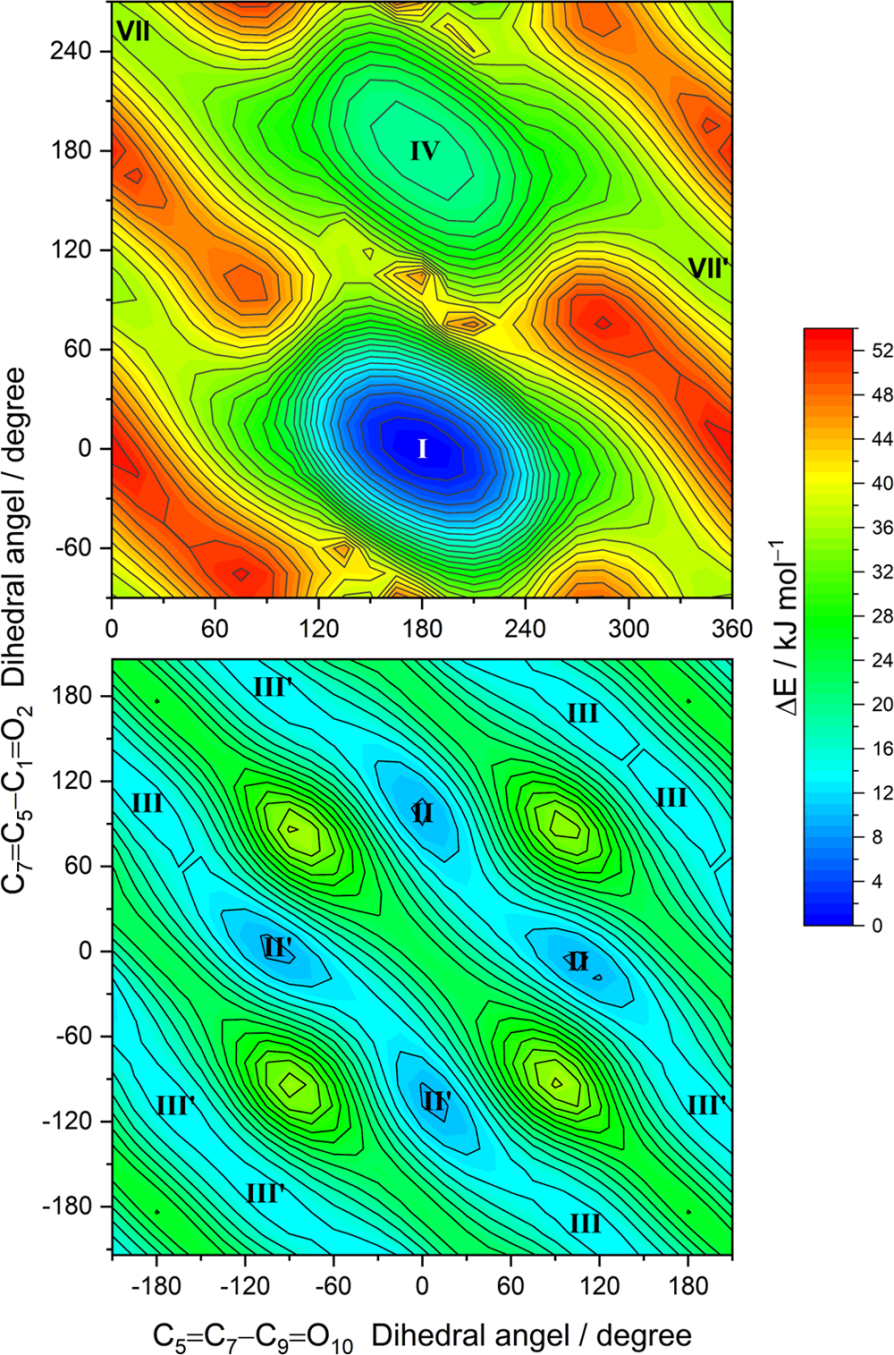
Relaxed potential energy surface contour maps of MA calculated at the DFT(B3LYP)/6-311++G(d,p) level. The C5=C7–C9=O10 and C7=C5–C1=O2 dihedral angles were incremented in steps of 15°, and all remaining internal coordinates were optimized at each point. The locations of conformers are indicated by the Roman numerals. The color bar designates the energy scale defined relative to the electronic energy of the lowest-energy form **I** (without the zero-point vibrational corrections). The isoenergy contour lines are traced using steps of 2 kJ mol^–1^.

In the molecule of maleic acid, there are four conformationally relevant degrees of freedom, which correspond to the internal rotations around the C–C bonds connecting the carboxylic groups to the central C=C bond and around the C–O bonds. Because of the proximity of the two carboxylic groups, interactions between them are strong, being either of an attractive nature (intramolecular H-bond) or a repulsive nature (H···H; O···O). This fact makes *a priori* predictions of which conformations should correspond to minima not easy. For example, if one assumes the different combinations of *cis* and *trans* carboxylic groups in a coplanar arrangement, then 10 different structures are obtained (3 *cis–cis*, 4 *cis–trans*, and 3 *trans–trans*). However, it is clear that some of these structures are highly strained because of strong repulsive H···H or/and O···O interactions, whereas nonplanar geometries shall exist that correspond to minimum-energy structures. To locate the minima, we performed relaxed 2D scans on the potential energy surface of the molecule where the scanned coordinates were the C_5_=C_7_–C_9_=O_10_ and C_7_=C_5_–C_1_=O_2_ dihedral angles. Three different scans were performed, one for each combination of *cis* and *trans* conformations of the carboxylic groups (i.e., *cis–cis*, *cis–trans*, and *trans–trans*). The scan maps using the DFT(B3LYP)/6-311++G(d,p) level for the first two cases are shown in Figure 1, where the locations of the minima are indicated by Roman numbers that correspond to the adopted designation for the conformers (or to that of their symmetry-related forms, indicated by the prime symbol after the Roman number).

**Figure 2:**
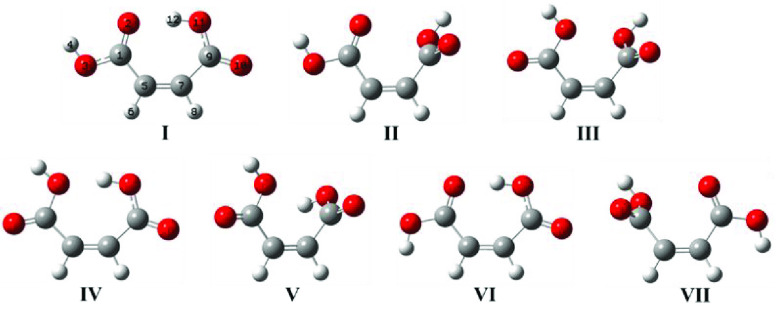
Optimized structures of maleic acid, including the numbering of atoms adopted in this work. At the DFT(B3LYP) level, structure **V** is not a minimum because it relaxes to conformer **IV** upon optimization. The MP2 method does not predict structures **VII** and **IV** as minima, when 6-311++G(d,p) and 6-311++G(2d,2p) are used, respectively. Colors: C, gray; H, white; and O, red.

The full set of calculations performed at the different levels of theory [DFT(B3LYP) and MP2, with the 6-311++G(d,p) and 6-311++G(2d,2p) basis sets] results in seven different putative minima (two *cis–cis* forms, four *cis–trans* forms, and one *trans–trans* form), which are displayed in Figure 2. Their relative energies are given in Table 1, while the optimized bond lengths and angles are provided in Table S1 (Supporting Information).

**Table 1:** Electronic Relative Energies (Including Zero-Point Corrections), in kJ mol^–1^, Calculated at the DFT(B3LYP) and MP2 Levels of Theory for the Conformers of MA and FA

conformers	DFT(B3LYP)/		MP2/		
maleic acid	6-311++G(d,p)	6-311++G(2d,2p)	6-311++G(d,p)	6-311++G(2d,2p)	symmetry
**I**	0.0	0.0	0.0	0.0	*C*_*s*_
**II**	8.0	10.1	0.1	3.6	*C*_1_
**III**	11.7	13.9	3.9	7.4	*C*_1_
**IV**	19.2	21.5	18.0		*C*_1_
**V**			20.1	22.0	C_1_
**VI**	24.3	22.0	26.6	22.5	*C*_*s*_
**VII**	33.3	33.3		27.2	*C*_1_
**fumaric acid**					
**I**	0.0	0.0	0.0	0.0	*C*_2*h*_
**II**	1.6	1.7	1.7	1.2	*C*_*s*_
**III**	2.7	2.8	2.5	2.2	*C*_2*h*_
**IV**	23.4	21.5	24.8	21.7	*C_s_*
**V**	26.3	24.5	27.4	24.1	*C*_*s*_
**VI**	29.3	27.3	29.4	27.1	*C*_1_
**VII**	30.3	28.1	30.1	27.7	*C*_1_
**VIII**	46.4	42.4	48.9	42.9	*C*_2*h*_
**IX**	57.4	53.2	58.0	53.2	*C*_1_
**X**	58.0	53.4	59.9	54.5	*C*_1_

Of the seven structures predicted by the calculations, form **V** is not a minimum at the DFT level (it relaxes to conformer **IV** upon optimization), while forms **IV** and **VII** are not minima when calculated at the MP2 level with the 6-311++G(d,p) and 6-311++G(2d,2p) basis sets, respectively. It shall be highlighted that when forms **IV**, **V**, and **VII** (all of them *cis–trans* species) are predicted as minima by the calculations, they correspond to high-energy forms, with relative energies above 18 kJ mol^–1^ (see Table 1). The *trans–trans* conformer **VI** is also predicted to have high relative energy (above 22 kJ mol^–1^) by the different combinations of method and basis set used. The *cis–trans* structure **I** and the two *cis–cis* forms **II** and **III** were predicted to be true low-energy minima by all calculations.

In agreement with previously reported data,^32^ the most stable conformer is the *cis–trans* form **I** (*C_s_* symmetry point group), bearing a strong OH···O= hydrogen bond (1.665 Å) that stabilizes this form compared to the others. The *cis–cis* conformers **II** and **III** (both nonplanar; *C*_1_) are 8.0–10.1 and 11.7–13.9 kJ mol^–1^ higher in energy than the most stable form, according to the DFT calculations, while the MP2 calculations yield relative energies for these two conformers that are considerably smaller (0.1–3.6 and 3.9–7.4 kJ mol^–1^, respectively; see Table 1). In conformer **II**, one of the carboxylic groups stays nearly in the main molecular plane and is oriented as in conformer **I**, being strictly planar, whereas the second carboxylic group is at almost perpendicular orientation in relation to the main molecular plane (C=C–C=O dihedral equal to 110.0°; DFT calculated value), with its O=C–O–H dihedral angle being −5.1°. The geometry of conformer **III** is similar to that of conformer **II**, but in conformer **III** the carboxylic group that stays nearly in the main molecular plane of the molecule is rotated by 180° relative to its orientation in conformer **II**. The orientation of the second carboxylic group in conformer **III** is identical to that found in conformer **II**, with the C=C–C=O dihedral equal to 110.0°, whereas the O=C–O–H dihedral angles in this conformer are −1.5 and −2.9° for in-plane and out-of-plane carboxylic groups, respectively.

In the *cis–trans* conformer **IV** (18.0–19.2 kJ mol^–1^), the *cis* carboxylic group is in the same orientation as the in-plane carboxylic group of **III**, whereas the *trans* carboxylic group assumes the same orientation as in **I** and acts as a donor in a weak OH···OH hydrogen bond (1.721 Å). This conformer has *C*_1_ symmetry but is nearly planar (see Figure 2). In turn, conformer **VII** (*C*_1_; 27.2–33.3 kJ mol^–1^) is structurally related to conformer **II**; nevertheless, the in-plane carboxylic group is *trans* instead of *cis*. Finally, the *trans–trans* conformer **VI** is planar (*C_s_* symmetry) and, like conformer **I**, is stabilized by a strong O–H···O= hydrogen bond (1.665 Å). Its energy is, however, 22.0–26.5 kJ mol^–1^ higher than that of conformer **I** as a result of the OH···H repulsion it bears and also because a non-hydrogen-bonded *trans*-carboxylic acid arrangement is (*per se*) considerably less stable than a *cis* arrangement.^50−52^ (These two destabilizing features are also present in conformer **VII** and determine its high relative energy too.)

Conformers **I**–**IV** and **VI** correspond to conformers **I**–**V** previously predicted by Maçôas et al.^32^ at the DFT(B3LYP)/6-31G(d,p) level (with relative energies of 0.0, 14.6, 18.7, 22.9, and 24.7 kJ mol^–1^, respectively). No *trans–trans* conformers were investigated in that previous work.

According to DFT(B3LYP)/6-311++G(d,p) calculations, the height of the energy barrier for the **I** → **VI** conformational conversion is predicted to be 52.0 kJ mol^–1^ (27.8 kJ mol^–1^ in the reverse direction), whereas those for the **II** → **III**, **III** → **IV**, and **II** → **VII** conversions are 14.7, 35.8, and 49.0 kJ mol^–1^, respectively (11.0, 28.3, and 23.7 kJ mol^–1^ for the reverse processes). As will be shown later in this article, the height of these barriers is relevant to the interpretation of the experimental data (conformational conversions induced by IR excitation and conformational decay in the dark via quantum mechanical tunneling).

#### 4.1.2 Fumaric Acid

**Figure 3:**
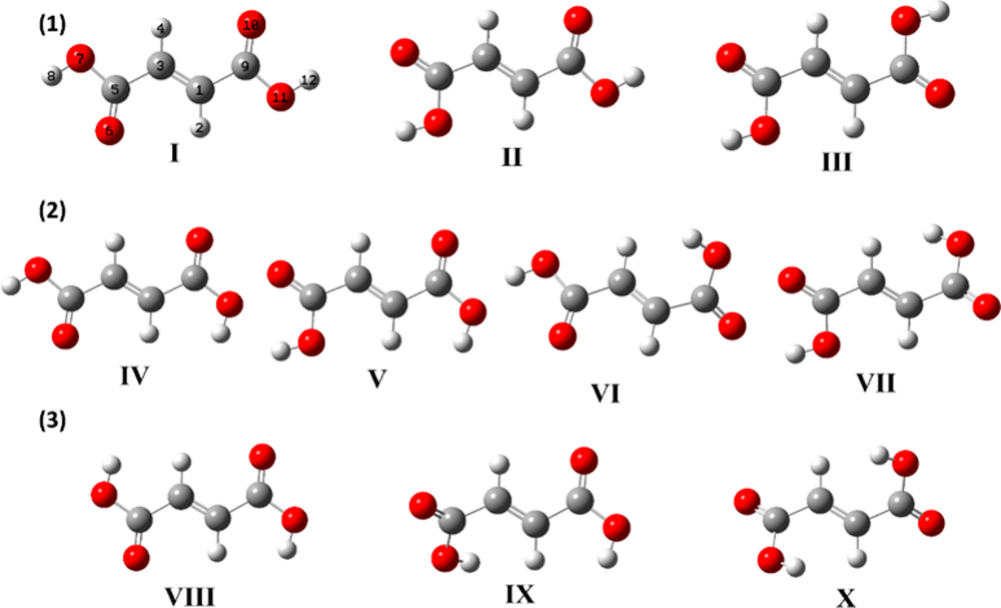
Optimized structures of the conformers of fumaric acid including the numbering of atoms adopted in this work. Colors: C, gray; H, white; and O, red.

**Figure 4:**
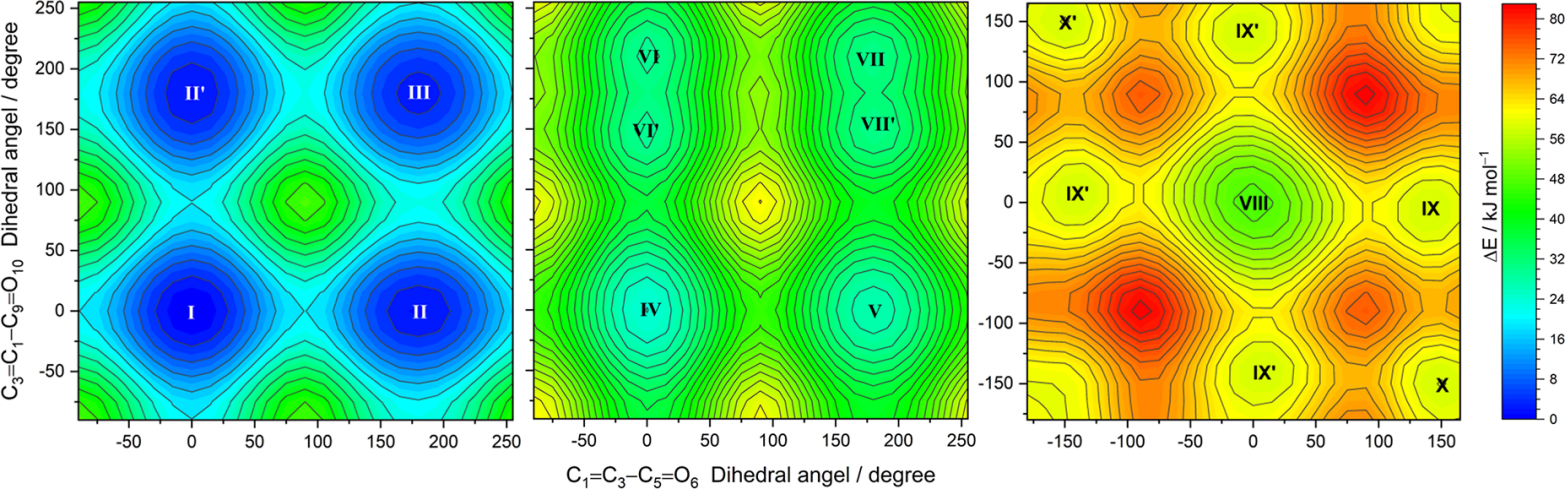
Relaxed potential energy surface contour maps of FA calculated at the DFT(B3LYP)/6-311++G(d,p) level for *cis–cis* (left panel), *cis–trans* (middle), and *trans–trans* (right) conformers. The C1=C3–C5=O6 and C3=C1–C9=O10 dihedral angles were incremented in steps of 15°, and all remaining internal coordinates were optimized at each point. The locations of six conformers are indicated by the Roman numerals. The color bar designates the energy scale defined relative to the electronic energy of the lowest-energy form **I** (without the zero-point vibrational corrections). The isoenergy contour lines are traced using steps of 2 and 4 kJ mol^–1^.

As in MA, fumaric acid has four conformationally relevant degrees of freedom, corresponding also to the internal rotations around the C–C and C–O bonds of the molecule. Because in fumaric acid the two carboxylic groups are placed on opposite sides of the double bond, they are far apart and do not interact with one another. Then, as could be anticipated, the calculations predict the existence of 10 different conformers, which correspond to the different combinations of *cis* and *trans* carboxylic groups (3 *cis–cis*, 4 *cis–trans*, and 3 *trans–trans*). These conformers are displayed in Figure 3, and the DFT(B3LYP)/6-311++G(d,p) 2D-scan maps for *cis–cis*, *cis–trans*, and *trans–trans* forms are shown in Figure 4, with the scanned coordinates being identical to those used for the construction of the 2D-scan maps of maleic acid shown in Figure 1. The relative energies of the conformers are provided in Table 1, and the bond lengths and angles are in Table S2 (Supporting Information).

The three *cis–cis* conformers (**I**–**III)** have the lowest energies among all conformers of FA because, as already mentioned, the *cis* geometry of a carboxylic group is intrinsically more stable than the *trans* geometry.^50−52^ All three conformers are planar (*C*_2*h*_, *C_s_*, and *C*_2*h*_ symmetry point groups, respectively), differing in the orientation of the carboxylic groups relative to the central C=C bond. As shown in Figure 4, they can be interconverted by rotations of 180° around the C–C bonds connecting the carboxylic groups to the C=C bond. In the most stable conformer **I**, both carboxylic groups are oriented in such a way that they form two O=C–C=C–H five-membered rings that are stabilized by CH···O= attractive interactions. In conformers **II** and **III**, one or both of the stabilizing O=C–C=C–H five-membered rings present in the most stable conformer are replaced by less-stabilizing CH···OH interactions. Accordingly, they are 1.2–1.7 and 2.2–2.7 kJ mol^–1^ higher in energy than conformer **I**, respectively.

The energies of the *cis–trans* conformers are intermediate (21.5–30.3 kJ mol^–1^ above that of conformer **I**). Conformer **IV** is the most stable of the *cis–trans* conformers, bearing two stabilizing O=C–C=C–H five-membered rings (as in conformer **I**; see Figure 3). Conformers **V** and **VI** (like conformer **II**) have only one stabilizing O=C–C=C–H five-membered ring. The energy of conformer **VI** is higher than that of conformer **V** because in form **VI** a stronger repulsive OH···H interaction exists compared to that present in conformer **V**. (This fact is reflected in the considerable out-of-plane geometry of the *trans* carboxylic group in **VI**; see Figure 3.) Conformer **VII** has no stabilizing O=C–C=C–H five-membered ring (like conformer **III**) and also has a strong repulsive OH···H interaction, being the higher-energy *cis–trans* conformer. The *cis–trans* conformers can be structurally related to the *cis–cis* forms because they differ from the latter by an ∼180° internal rotation around one of the two C–O bonds of the molecule: conformer **IV** (*C_s_* symmetry) is related to conformer **I**, conformers **V** (*C_s_*) and **VI** (*C*_1_) are related to conformer **II**, and conformer **VII** (*C*_1_) is related to conformer **III**.

The three *trans–trans* conformers (**VIII**–**X**) are the highest-energy forms (42.4–59.9 kJ mol^–1^). These conformers can be obtained from conformers **I**–**III**, respectively, by internal rotation around both C–O bonds, and are of *C*_2*h*_ (**VIII**) and *C*_1_ (**IX** and **X**) symmetry. Their relative energies can be easily explained by the same factors used above to explain the relative energies of the *cis–cis* and of the *cis–trans* conformers.

In the previous study of Maçôas et al.,^32^ only five conformers of FA were described, which correspond to forms **I**–**V** (the three *cis–cis* conformers and the two lowest energy *cis–trans* forms). The relative energies calculated in that previous study are consistent with our results, with the relative energies of conformers **II** and **III** being predicted to be within 5 kJ mol^–1^ and those of conformers **IV** and **V** being *ca*. 25 kJ mol^–1^.^32^ On the other hand, Vogt et al.^22^ investigated the whole set of structures reported in our study, but only six of these (**I**–**VI**) appeared to be minimum-energy structures at the MP2/cc/pVTZ level of theory. However, as will be shown later in this article, fumaric acid conformer **VII** could be observed experimentally in the present work, which doubtlessly demonstrates that this (and probably all of the remaining forms predicted in our calculations) is indeed a true conformational state of FA.

The transformations between conformers belonging to each group of conformers of FA (*cis–cis*, *cis–trans*, and *trans–trans*) involve the movement of heavy atoms. The energy barrier heights for these conformational conversions are relatively low. The barriers associated with the conversions between the FA lowest-energy *cis–cis* conformers, **I** → **II** and **II** → **III** transformations, are predicted by the DFT(B3LYP)/6-311++G(d,p) calculations to be equal to 19.3 kJ mol^–1^ (17.7 kJ mol^–1^ in the reverse direction) and 19.8 kJ mol^–1^ (18.7 kJ mol^–1^ in the reverse direction), respectively. On the other hand, the conversions between conformers belonging to distinct groups involve mostly movements of the light hydrogen atoms, making these processes more probable under low-temperature matrix isolation conditions because they may take place by quantum mechanical tunneling.^53,54^ The predicted energy barrier for the **I** → **IV** conversion is 53.6 kJ mol^–1^ (30.2 kJ mol^–1^ in the reverse direction), whereas that for the **III** → **VII** transformation amounts to 50.0 kJ mol^–1^ (22.4 kJ mol^–1^ for the reverse reaction). In turn, the **II** → **V** and **II** → **VI** conversions have associated barriers of 50.2 and 46.7 kJ mol^–1^, respectively (25.5 and 19.0 kJ mol^–1^ in the opposite direction), and those for the **IV** → **VIII**, **VI** → **IX**, and **VII** → **X** conversions are 50.0, 51.8, and 46.0 kJ mol^–1^, respectively (43.2, 18.3, and 23.7 kJ mol^–1^ for the inverse transformations).

### 4.2 Infrared Spectra of Matrix-Isolated Maleic Acid

#### 4.2.1 Infrared Spectra of Conformers **I** and **II**

**Figure 5:**
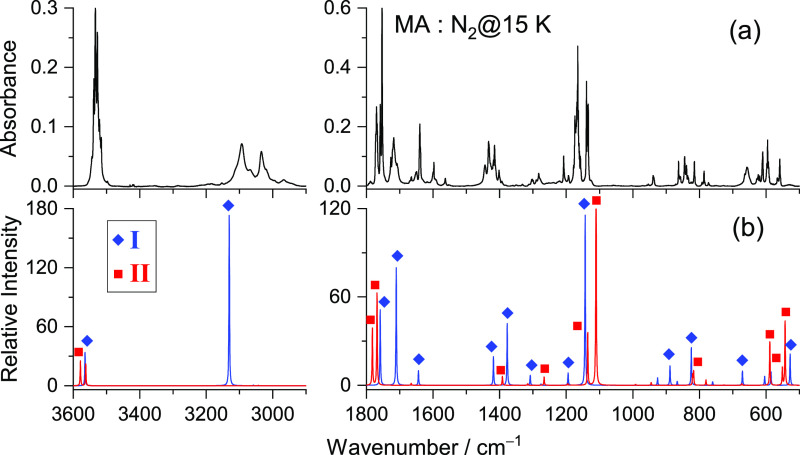
Experimental FTIR spectrum of MA isolated in a N_2_ matrix at 15 K after deposition (a) and theoretical infrared spectra with the anharmonic frequencies of conformers **I** (blue) and **II** (red) (b) calculated at the DFT(B3LYP)/6-311++G(d,p) level.

Figure 5 displays the mid-IR spectrum of MA isolated in solid nitrogen at 15 K, together with the calculated anharmonic spectra of the two most stable conformers (**I** and **II**). The comparison of the experimental and calculated spectra indicates that only these two conformers are present in the as-deposited matrix. The assignments of the fundamental bands for conformers **I** and **II** are given in Tables 2 and 3, respectively. The assignment also took into account the results of the selective vibrational excitation experiments and tunneling kinetics described in Sections 4.2.2–4.2.3. The anharmonic vibrational frequencies and intensities of the fundamental modes for all of the conformers are given in Table S3 (Supporting Information).

**Table 2:** Experimental (in N_2_ and Ar Matrixes) and DFT(B3LYP)/6-311++G(d,p) Calculated Infrared Data for Conformer **I** of MA with the Proposed Assignments^a^

	experimental		calculated	
approximate description	N_2_ matrix	Ar matrix^32^	ν	*I*^IR^
ν(OH)1	3533.5	3550	3564.6	107
ν(OH)2	3154.0/3093.5/3034.0	3110	3130.9	548
ν(CH)s	n. obs.	n. obs.	3058.6	2
ν(CH)a	n. obs.	n. obs.	3004.2	<1
ν(C=O)2	*1753.0*	1762/1750/1740	1758.6	164
ν(C=O)1	1727.0/1722.0/1718.0	1722/1712	1710.5	254
ν(C=C) + ν(CC)1 + δ(CCH)1	1639.5	1635	1643.8	31
δ(CCH)1 + δ(CCH)2	1444.0/1433.0/*1432.0*	1428/*1420*	1418.7	63
δ(COH)2 + δ(HC=C)1 + ν(CO)2	1402.0	1410/1406/1403	1377.3	134
δ(COH)1 + δ(C=CH)2 + δ(CCH)2	1283.0	1313	1308.2	22
δ(C=CH)1 + δ(CCH)2	n. obs.	1296	1275.9	3
δ(CCH)1 + ν(CO)2 + δ(C=CH)2	1208.0	1174	1194.4	26
δ(COH)1 + ν(CO)1 + δ(HC=C)1	1168.0/1165.0/*1162.0*/1157.5	*1154*/*1152*	1143.2	371
γ(CH)a + τ(C=C)	n. obs.	1027/1025	1071.9	1
ν(CC)1 + δ(CCH)2 + δ(CC=C)	954.0/*939.0*	933	925.8	16
ν(CC)1 + γ(CH)s + γ(C=O)2	863.0	857	888.4	42
ν(CC)1 + ν(CC)2 + δ(CC=O)2	844.0	822	866.7	9
γ(OH)2 + τ(C=C) + γ(C=O)1	839.0/833.0	782	824.5	80
δ(CCC)2 + δ(C=CC)2	n. obs.	775	815.7	0
τ(CO)2 + γ(OH)2	773.0	767	760.4	8
τ(CO)1 + γ(OH)1	657.5	*631*	671.0	31
δ(OCO)1 + δ(OCO)2	610.0	606	604.2	19
δ(OCO)2 + δ(OCO)1 + ν(CC)1	597.0/595.0/593.0	589	585.1	29
γ(OH)1 + τ(CC)2	571.5/567.0/566.0/*563.0*/*559.0*/556.5	552	527.7	68
δ(CC=O)2 + δ(CC=O)1	n. i.	n. i.	388.1	1
δ(CC=O)1 + δ(CC=C) + δ(C=CC)	n. i.	n. i.	300.1	9
τ(C=C) + τ(CC)2 + τ(CC)1	n. i.	n. i.	283.0	8
δ(OCC)1 + δ(CC=C)	n. i.	n. i.	232.7	7
τ(CO)2 + τ(CC)1	n. i.	n. i.	86.8	1
τ(CC)2	n. i.	n. i.	38.2	3

a Wavenumbers (cm^–1^); calculated intensities (km mol^–1^); s = symmetric; a = antisymmetric; ν = stretching; δ = in-plane bending; γ = out-of-plane bending; τ = torsion; n. obs. = not observed; and n. i. = not investigated. 1 and 2 refer to the *cis* and *trans* carboxylic groups, respectively. Frequency values shown in *italic* are assigned to more than one conformer.

**Table 3:** Experimental (in N_2_ and Ar Matrixes) and DFT(B3LYP)/6-311++G(d,p) Calculated Infrared Data for Conformer **II** of MA with the Proposed Assignments^a^

	experimental		calculated	
approximate description	N_2_ matrix	Ar matrix^32^	ν	*I*^IR^
ν(OH)1	3538.0	3566	3578.5	80
ν(OH)2	3527.5/3521.0	3561	3562.5	70
ν(CH)1 + ν(CH)2	n. obs.	n. obs.	3043.1	1
ν(CH)2 + ν(CH)1	n. obs.	n. obs.	3009.6	<1
ν(C=O)2	1770.0	1808/1780/1790/1783/1778/1773/1769	1782.2	124
ν(C=O)1	1758.5	1761/1754	1768.3	201
ν(C=C)	1649.5	1698/1570	1665.6	4
δ(CCH)1 + δ(C=CH)1 + δ(C=CH)2	1420.0/1419.0	*1420*	1391.7	20
δ(COH)2	1350.0	1370	1315.9	3
δ(COH)1 + δ(C=CH)2 + δ(CCH)2	n. obs.	1290	1266.4	18
δ(CCH)1 + δ(CCH)2 + δ(C=CH)1 + δ(C=CH)2	1193.5/1192.0	1163	1195.5	3
ν(CO)2 + δ(COH)2	1174.0/*1162.0***/**1161.0	1150/1147	1135.5	113
ν(CO)1 + δ(COH)1	1139.0/1137.0/1134.0	1122	1110.5	467
γ(CH)a + τ(C=C)	n. obs.	n. obs.	991.7	2
δ(C=CH)2 + δ(CCH)2	952.0/*939.0*	934	945.0	6
ν(CC)1 + ν(CC)2 + τ(CO)1	831.0	828	817.5	30
γ(C=O)1 + γ(CH)s + τ(CC)2	829.0/815.0	828	820.5	22
γ(C=O)2 + τ(CO)2	792.0/786.0	811	780.4	12
γ(CH)1 + τ(CC)1 + τ(CO)2	n. obs.	781	726.1	1
γ(OH)2 + τ(CO)2	n. obs.	*631*	672.7	3
γ(OH)1 + τ(CO)1	628.0/623.5	591	588.7	95
γ(OH)2 + τ(CO)2	597.0/*592.0*/590.0	568	551.1	38
δ(CC=O)2 + ν(CC)2	*563.0***/***559.0*	549	542.8	138
γ(OH)2 + τ(CO)2	n. i.	n. i.	459.6	3
γ(OH)1 + τ(C=C)1 + γ(CH)2	n. i.	n. i.	435.1	30
δ(CC=O)1 + δ(C=CC) + γ(OH)2 + τ(CC)2	n. i.	n. i.	284.5	1
τ(CC)1 + τ(C=C) + τ(CC)2	n. i.	n. i.	233.8	2
δ(CC=C) + δ(C=CC) + τ(CC)2	n. i.	n. i.	126.0	2
τ(CC)1 + τ(CC)2	n. i.	n. i.	79.5	2
τ(CC)1 + τ(CC)2	n. i.	n. i.	24.8	1

a Wavenumbers (cm^–1^); calculated intensities (km mol^–1^); s = symmetric; a = antisymmetric; ν = stretching; δ = in-plane bending; γ = out-of-plane bending; τ = torsion; n. obs. = not observed; and n. i. = not investigated. 1 and 2 refer to the in-plane and out-of-plane carboxylic groups, respectively. Frequency values shown in *italic* are assigned to more than one conformer.

With the exception of a few low-intensity bands, it was possible to identify all of the experimental bands belonging to conformers **I** and **II**.

The most intense bands (or groups of bands) assigned to conformer **I** appear at 3533.5, 1753.0, 1727.0/1722.0/1718.0, 1168.0/1165.0/1162.0/1157.5, and 863.0 cm^–1^, which are assigned to the free OH stretching mode ν(OH)1, the carbonyl stretching modes ν(C=O)2 and ν(C=O)1, and two vibrations described as δ(COH)1 + ν(CO)1 + δ(HC=C)1 and ν(CC)1 + γ(CH)s + γ(C=O)2, respectively. The OH stretching mode of the group involved in the intramolecular hydrogen bond is predicted to be at 3130.9 cm^–1^ (with the highest intensity of 548 km mol^–1^) and is observed as a broad band with relative maxima at 3154.0/3093.5/3034.0 cm^–1^. The bands observed for MA in the argon matrix by Maçôas et al.^32^ at 1296, 1027/1025, and 775 cm^–1^, due to the ν(CO)2, γ(CH)a, and δ(CCC) vibrations, are not observed in the N_2_ matrix (see Table 2).

For conformer **II**, the most characteristic bands are observed at 3538.0 and 3527.5 cm^–1^, ascribed to the two OH stretching modes (ν(OH)1 and (ν(OH)2), respectively); at 1770.0 and 1758.5 cm^–1^, assigned to the C=O stretching modes (ν(C=O)2 and ν(C=O)1); in the 1194–1134.0 cm^–1^ spectral region, corresponding to the three composed modes described as δ(CCH)1 + δ(CCH)2 + δ(C=CH)1 + δ(C=CH)2, ν(CO)2 + δ(COH)2, and ν(CO)1 + δ(COH)1; and at 597.0/592.0/590.0 and 563.0/559.0 cm^–1^, which are due to the γ(OH)2 + τ(CO)2 and δ(CC=O)2 + ν(CC)2 vibrational modes. All of these bands have counterparts in the spectrum obtained for MA isolated in solid argon^32^ (see Table 3).

#### 4.2.2 Conformers **VI** and **VII**: Selective NIR Irradiation at 6898.5 and 6883.5 cm^–1^

Narrowband selective vibrational excitation of the first OH stretching overtone vibrations of the two conformers of MA initially present in the N_2_ matrix (**I** and **II**) was used to successfully generate the higher-energy conformers **VI** and **VII**.

Figure S1 (Supporting Information) shows a fragment of the NIR experimental spectrum of MA isolated in a N_2_ matrix at 15 K, compared with the anharmonic simulated spectra of conformers **I** and **II** in the NIR spectral region. The experimental bands observed at 6898.5 and 6883.5 cm^–1^ are assigned to the first OH stretching overtone vibrations of conformers **I** and **II**, respectively, whose fundamentals appear at 3533.5 and 3538.0/3527.5 cm^–1^.

**Scheme 1:**
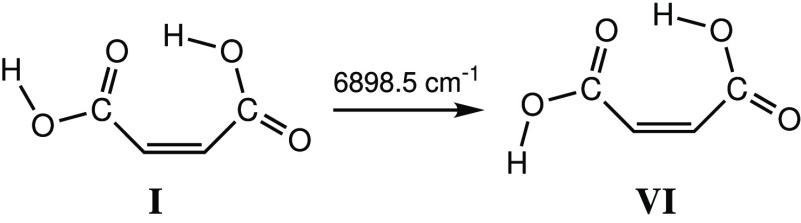
Narrowband Vibrational Excitation of the First OH Stretching Overtone at 6898.5 cm^–1^ of Conformer **I** of MA and the Formation of Conformer **VI**

Excitation at 6898.5 cm^–1^ (**I**) led to an intensity reduction of the bands belonging to conformer **I** by ca. 69%, while new bands emerged in the spectrum, indicating the generation of a new conformer. Comparison of the calculated spectra for the conformers of MA with the experimental spectrum emerging upon the performed irradiation led to the conclusion that the resulting species corresponds to conformer **VI**, which differs from conformer **I** by internal rotation of the free OH group (Scheme 1).

**Figure 6:**
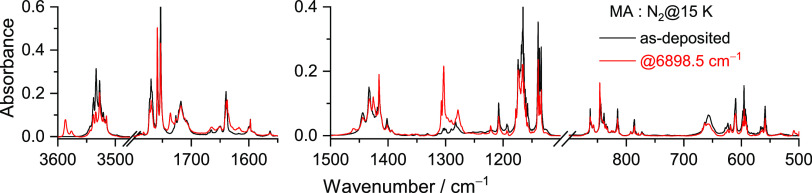
Experimental FTIR spectra of MA isolated in a N_2_ matrix at 15 K after deposition (black) and after irradiation at 6898.5 cm^–1^ for 120 min (red).

Figure 6 shows the infrared spectra obtained before and after irradiation at 6898.5 cm^–1^. Note that the bands of conformer **II**, also present in the nonirradiated matrix as pointed out in the previous section, remained unchanged upon this irradiation. Table 4 lists the assignments of the observed fundamental bands of conformer **VI**, which are compared with the corresponding calculated anharmonic vibrational frequencies and intensities. The most characteristic bands of conformer **VI** are observed at 3587.0/3576.0, 1736.0, 1426.0, 1307.0/1303.0/1278.5, and 779.0 cm^–1^, which correspond to ν(OH)1, a mixed vibration associated with the stretching of the two carbonyl groups (ν(C=O)2 + ν(C=O)1), and the composing vibrations described as δ(CCH)1 + δ(COH)2, δ(COH)1 + ν(CO)1, and δ(OCC)1 + δ(CC=C) + δ(C=CC), respectively (see Table 4).

**Table 4:** Experimental (in the N_2_ Matrix) and DFT(B3LYP)/6-311++G(d,p) Calculated Infrared Data for Conformer **VI** of MA with the Proposed Assignments^a^

	experimental	calculated	
approximate description	N_2_ matrix	ν	*I*^IR^
ν(OH)1	*3587.0***/***3576.0*	3620.8	64
ν(OH)2	2992.0	3075.6	537
ν(CH)2 + ν(CH)1	n. obs.	3064.3	3
ν(CH)1 + ν(CH)2	2966.0/2958.0	2965.2	6
ν(C=O)2 + ν(C=O)1	1736.0	1768.6	216
ν(C=O)1 + ν(C=O)2	n. obs.	1727.0	187
ν(C=C) + δ(CCH)1 + δ(CCH)2	1618.0	1626.3	52
δ(CCH)1 + δ(COH)2	1426.0	1383.5	150
δ(CCH)1 + δ(CCH)2 + δ(COH)2	1461.0	1420.3	16
δ(CCH)2 + ν(CO)2	1290.0	1288.1	1
δ(COH)1 + ν(CO)1	1307.0/1303.0/1278.5	1257.4	545
δ(CCH)1 + δ(C=CH)2 + ν(CO)2	1222.0	1199.8	27
δ(COH)1 + ν(CO)2 + δ(CCH)2	n. obs.	1129.3	4
γ(CH)a + τ(C=C)	n. obs.	1069.0	<1
ν(CC)1 + δ(CC=C)1 + δ(CCH)2	942.0	929.2	12
γ(CH)s + γ(OH)2 + τ(CO)2	846.0	901.4	72
ν(CC)1 + ν(CC)2 + δ(OCO)2	n. obs.	849.9	<1
γ(OH)2 + τ(CC)2 + γ(C=O)1	n. obs.	821.8	10
δ(OCC)1 + δ(CC=C) + δ(C=CC)	779.0	769.8	64
γ(OH)2 + τ(CC)1 + τ(C=C) + τ(CC)2	n. obs.	760.7	4
δ(OCC)2 + δ(CC=O)1 + ν(CC)1	618.0	610.9	4
δ(OCC)1 + δ(OCO)2 + ν(CC)2 + δ(CC=O)2	n. obs.	591.0	1
γ(OH)1 + γ(CH)s + γ(C=O)2	n. obs.	585.0	4
γ(OH)1	532.0/509.0	486.3	99
δ(CC=O)2 + δ(OCC)1	n. i.	386.4	6
δ(CC=C)1 + δ(C=CC)2 + δ(CC=O)2	n. i.	290.3	<1
τ(C=C) + τ(CC)2 + τ(CC)1	n. i.	277.7	3
δ(CC=C)1 + δ(OCC)1 + δ(C=CC)2	n. i.	236.7	26
τ(CC)2 + τ(CO)2	n. i.	78.0	4
τ(CC)1 + τ(CC)2	n. i.	18.3	4

a Wavenumbers (cm^–1^); calculated intensities (km mol^–1^); s = symmetric; a = antisymmetric; ν = stretching; δ = in-plane bending; γ = out-of-plane bending; τ = torsion; n. obs. = not observed; and n. i. = not investigated. 1 and 2 refer to the acceptor and donor H-bonded carboxylic groups, respectively. Frequency values shown in *italic* are assigned to more than one conformer.

**Scheme 2:**
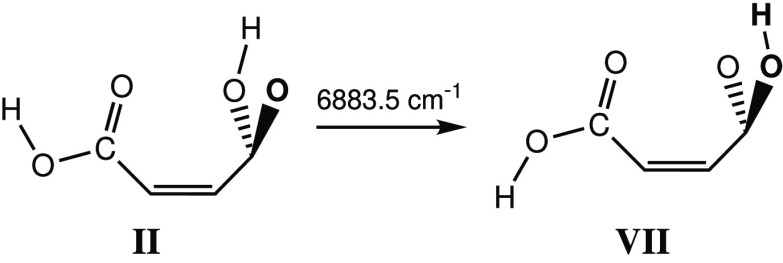
Narrowband Vibrational Excitation of the First OH Stretching Overtone at 6883.5 cm^–1^ of Conformer **II** of MA and the Formation of Conformer **VII**

**Figure 7:**
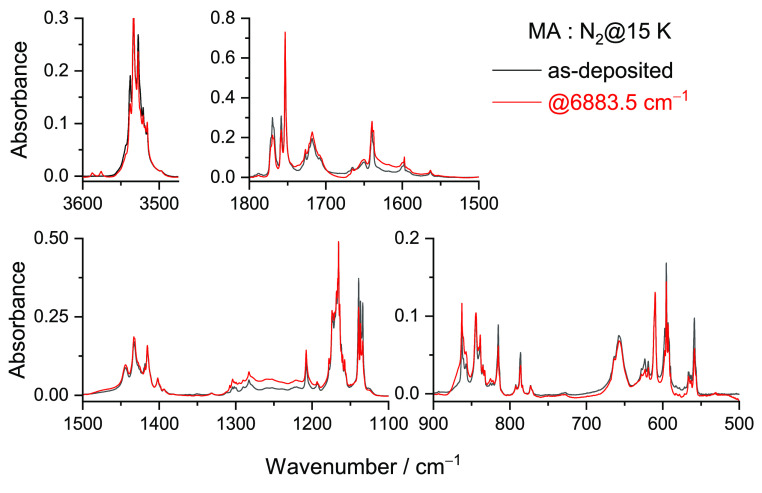
Experimental FTIR spectra of MA isolated in a N_2_ matrix at 15 K after deposition (black) and after irradiation at 6883.5 cm^–1^ for ∼4 h (red).

On the other hand, the vibrational excitation of conformer **II** at 6883.5 cm^–1^ efficiently produced another set of new bands (Figure 7), which were assigned to conformer **VII** (Scheme 2).

Table 5 displays the assignment of the observed fundamental bands of conformer **VII**, together with the calculated anharmonic vibrational frequencies and intensities for this form. The most significant bands confirming the assignment of conformer **VII** are those observed at 3587.0/3576.0 and 1759.0 cm^–1^ as well as the multiplet at 1308.0/1304.5/1300.0/1296.0/1278.0 cm^–1^, which are ascribed to the ν(OH)1, ν(C=O)2, and δ(COH)1 + ν(CO)1 modes, respectively.

**Table 5:** Experimental (in the N_2_ Matrix) and DFT(B3LYP)/6-311++G(d,p) Calculated Infrared Data for Conformer **VII** of MA with the Proposed Assignments^a^

	experimental	calculated	
approximate description	N_2_ matrix	ν	*I*^IR^
ν(OH)1	*3587.0***/***3576.0*	3621.1	45
ν(OH)2	3516.0	3560.3	74
ν(CH)2 + ν(CH)1	n. obs.	3045.5	1
ν(CH)1 + ν(CH)2	n. obs.	2982.7	7
ν(C=O)1	1783.0	1792.0	21
ν(C=O)2	1759.0	1781.5	289
ν(C=C)	1638.0	1655.4	25
δ(CCH)1 + δ(C=CH)1 + δ(C=CH)2	1432.0	1404.8	30
δ(C=CH)2 + δ(COH)2	n. obs.	1312.2	7
δ(COH)1 + ν(CO)1	1308.0/1304.5/1300.0/1296.0/1278.0	1261.0	120
δ(CCH)1 + δ(CCH)2 + δ(C=CH)1 + δ(C=CH)2	1122.0	1202.3	4
δ(COH)2 + ν(CO)2	n. obs.	1135.7	245
δ(COH)1 + ν(CO)1	n. obs.	1096.2	27
γ(CH)a + τ(C=C)	n. obs.	967.6	1
δ(CC=C)1 + δ(C=CC)2	937.0	939.7	25
ν(CC)1 + ν(CC)1 + τ(CC)2	825.0	823.3	15
γ(C=O)1 + γ(CH)s + τ(CC)1	796.0	800.1	21
γ(C=O)2 + τ(CO)2 + τ(CC)1	n. obs.	779.5	13
γ(CH)1 + τ(C=C)	n. obs.	710.2	2
γ(OH)2 + τ(CO)2	n. obs.	672.8	9
γ(OH)2 + τ(CO)2	n. obs.^b^	540.9	81
δ(CC=O)2	531.0	555.3	26
γ(OH)1 + τ(CO)1	n. i.	519.5	4
γ(OH)2	n. i.	454.3	14
γ(OH)1	n. i.	314.1	71
γ(OH)2 + δ(CC=O)1 + δ(OCO)1	n. i.	289.8	8
τ(CC)1 + τ(C=C) + τ(CC)2	n. i.	230.2	9
τ(CC)2	n. i.	130.7	9
τ(CC)2 + γ(C=O)2	n. i.	83.6	4
τ(CC)1 + τ(CC)2	n. i.	15.0	1

a Wavenumbers (cm^–1^); calculated intensities (km mol^–1^); s = symmetric; a = antisymmetric; ν = stretching; δ = in-plane bending; γ = out-of-plane bending; τ = torsion; n. obs. = not observed; and n. i. = not investigated. 1 and 2 refer to the *trans* and *cis* carboxylic groups, respectively. Frequency values shown in *italic* are assigned to more than one conformer.

b Superimposed on the bands of conformer **II**.

**Figure 8:**
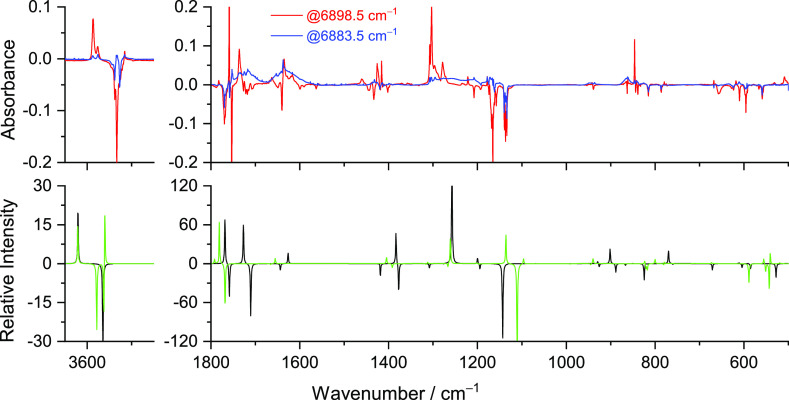
Experimental difference FTIR spectra of MA after irradiation at 6898.5 cm^–1^ for 120 min in a N_2_ matrix at 15 K (red) and after irradiation at 6883.5 cm^–1^ for ∼4 h (blue) minus the spectra of the freshly deposited MA (top). Simulated difference infrared spectrum with the anharmonic frequencies of conformer **VI** minus conformer **I** (black) and of conformer **VII** minus conformer **II** (green) (bottom) calculated at the DFT(B3LYP)/6-311++G(d,p) level.

Figure 8 shows a global picture of the changes produced in the matrix after NIR irradiation (at 6898.5 and 6883.5 cm^–1^), allowing the comparison of the experimental difference spectra (obtained by subtracting the as-deposited spectrum of MA from the spectra obtained after the performed NIR irradiation) with the simulated difference spectrum (**I** minus **VI**; **II** minus **VII**), which unequivocally confirms the existence of two distinct species consumed (forms **I** and **II**) and the respective products (forms **VI** and **VII**).

**Figure 9:**
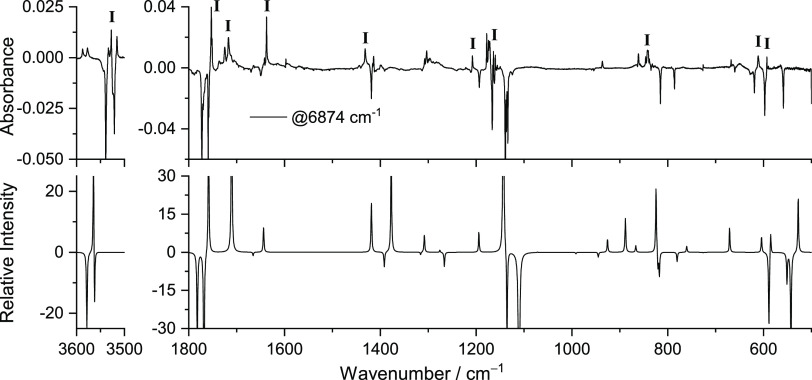
Experimental difference FTIR spectrum of MA after irradiation at 6974.0 cm^–1^ for 1.5 h minus the spectrum of the freshly deposited MA in a N_2_ matrix at 15 K (top). Bands belonging to conformer **I** are identified in the spectrum for better visualization. Simulated difference infrared spectrum with the anharmonic frequencies of conformer **I** minus conformer **II** (bottom) calculated at the DFT(B3LYP)/6-311++G(d,p) level.

Other irradiation was carried out. Irradiation at 6920.0 and 6904.5 cm^–1^ proved to induce only very minor spectral changes or none. On the other hand, irradiation at 6874.0 cm^–1^ (Figure 9) led to the consumption of conformer **II**, as the bands at 3538.0, 3521.0/3518.0, 1770.0, 1758.5, 1649.5, 1420.0/1419.0, 1193.5, 1139.0/1137.0/1134.0, 835.0, 815.0, 786.0, 619.0, 597.0, and 559.0 cm^–1^ assigned to this conformer were bleached. Simultaneously, an increase in the intensity of the bands belonging to conformer **I** was observed throughout the spectra (i.e., at 3527.5, 1753.0, 1718.0, 1639.5, 1433.0/1432.0, 1208.0, 1165.0, 845.0, 611.0/610.0, and 593.0 cm^–1^). This indicates that irradiation at 6874.0 cm^–1^ led mainly to the conversion of form **II** into the most stable form **I**. However, some new bands were also produced during this irradiation, which can be related to conformer **VII** (3587.0/3576.0, 1637.5, 1307.5, and 937.0 cm^–1^), indicating that the **II** → **VII** conversion also takes place as a minor process upon irradiation at 6874.0 cm^–1^.

Conformers **VI** and **VII** have never been previously observed experimentally. In the work of Maçôas et al.,^32^ narrowband tunable NIR irradiation at the first OH stretching overtone of conformer **II** (6901 cm^–1^) was found to promote only the conversion of conformer **II** to conformer **I** (a conversion that was also found to take place in that medium upon annealing of the matrix).^32^ The observation of high-energy conformers **VI** and **VII** in the present investigation confirms once again the ability of solid N_2_ to stabilize high-energy conformers of carboxylic acids bearing *trans* carboxylic groups because of the establishment of a stabilizing H-bond-type interaction between the OH group and the matrix N_2_ molecules.^55,56^

#### 4.2.3 Tunneling Reactions: **VI** → **I** and **VII** → **II** Decays in the Dark

Once formed as described in the previous section, the amount of conformers **VI** and **VII** was found to slowly decrease in the dark, with the simultaneous increase in the amount of conformers **I** and **II**, respectively. These conversions take place by the internal rotation of a free OH group, which involves the motion of the hydrogen atom only. Considering the temperature of the matrix and the size of the **VI** → **I** (27.8 kJ mol^–1^) and **VII** → **II** (23.7 kJ mol^–1^) barriers, the processes can occur only via hydrogen atom tunneling through the torsional barrier.^53,54,57^

**Figure 10:**
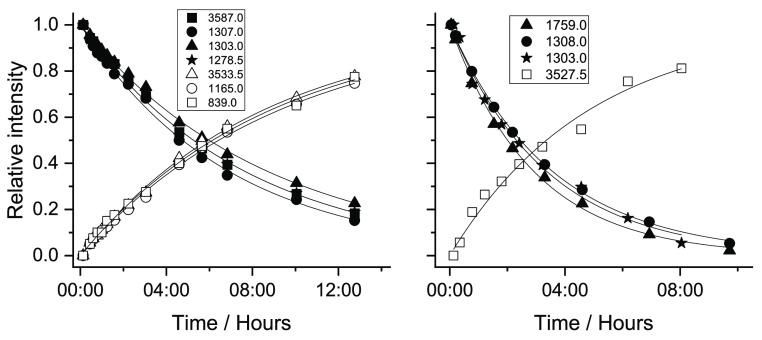
Tunneling decays of conformers **VI** and **VII** and the formation of conformers **I** and **II**, respectively, in a N_2_ matrix at 15 K after irradiation at 6898.5 and 6883.5 cm^–1^ (MA). The decay of **VI** was evaluated using bands at 3587.0 (ν(OH)1), 1307.0, 1303.0, and 1278.5 cm^–1^ (δ(COH)1 + ν(CO)1) and that of **VII** using bands at 1759.0 (ν(C=O)2), 1308.0, and 1304.5 cm^–1^ ((δ(COH)1 + ν(CO)1)). The recovery of conformer **I** was evaluated using the bands at 3533.5 (ν(OH)1), 1165.0 (δ(COH)1 + ν(CO)1 + δ(HC=C)1), and 839.0 cm^–1^ (γ(OH)2 + τ(C=C) + γ(C=O)1) and that of **II** using the band at 3527.5 cm^–1^ (ν(OH)2. The results were fitted with single-exponential functions.

The study of the tunneling decay was performed in the dark, exposing the sample to the radiation of the globar source of the spectrometer only during spectra recording. Figure 10 shows the kinetics of the tunneling decay of conformers **VI** and **VII** and the reformation of conformers **I** and **II**, respectively, as determined by using different bands. The lifetimes of conformers **VI** and **VII** in the N_2_ matrix (average value obtained from the different curves shown in Figure 10) were found to be about 5 h 30 min and ca. 2 h. The longer lifetime of conformer **VI**, compared to that of conformer **VII**, can be explained by the combination of the lower energy of **VI** and the higher barrier associated with the isomerization of this form.

### 4.3 Infrared Spectra of Matrix-Isolated Fumaric Acid

#### 4.3.1 Infrared Spectra of Conformers **I**–**III**

**Figure 11:**
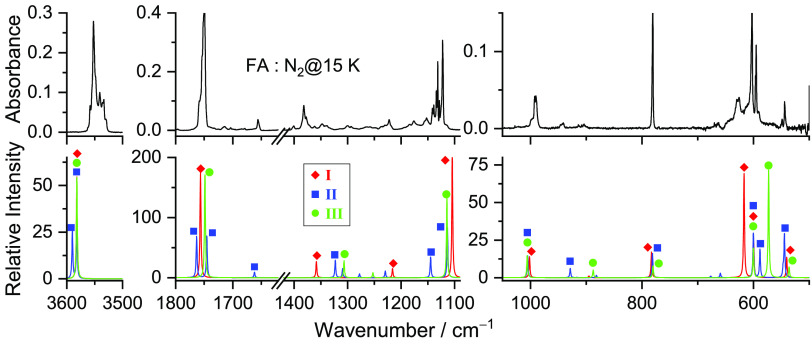
Experimental FTIR spectrum of FA isolated in a N_2_ matrix at 15 K after deposition (upper panel) and theoretical infrared spectra with the anharmonic frequencies of conformers **I** (red), **II** (blue), and **III** (green) (lower panel) calculated at the DFT(B3LYP)/6-311++G(d,p) level.

The IR spectrum of FA isolated in solid nitrogen at 15 K, together with the theoretical anharmonic spectra of the three most stable conformers (**I**–**III**), is presented in Figure 11. The comparison of the experimental and calculated spectra indicates that the three most stable conformers of FA are present in the as-deposited matrix. The assignments of the fundamental bands for conformers **I**–**III** are given in Tables 6–8. The anharmonic vibrational frequencies and intensities of the fundamental modes for all of the conformers of FA are provided in Table S4 (Supporting Information). The proposed assignments took into account the results of the selective vibrational excitation and tunneling kinetics experiments described in Sections 4.3.2–4.3.4.

**Table 6:** Experimental (in N_2_ and Ar Matrixes) and DFT(B3LYP)/6-311++G(d,p) Calculated Infrared Data for Conformer **I** of FA with the Proposed Assignments^a^

	experimental		calculated	
approximate description	N_2_ matrix	Ar matrix^32^	ν	*I*^IR^
ν(OH)a	*3558.0***/***3552.0*	*3560*	3582.0	174
ν(CH)s	3074.5	*2948*	3078.9	<1
ν(C=O)a	*1756.0***/***1754***/***1749.5*	*1765***/***1763***/***1759***/***1756***/***1754***/***1750*	1756.5	561
δ(COH)a	1401.0	1369	1358.3	86
δ(C=CH)a + δ(COH)a	1222.0	1217/1211	1216.0	49
δ(COH)a	1122.5	1115/1111	1104.4	652
γ(CH)a + τ(C=C)	*992.0***/***990.0***/***989.0*	982	1001.7	45
ν(CC)a + ν(CO)a	n. obs.	914	895.1	4
γ(C=O)s + γ(OH)s	*780.5*	777	782.9	54
γ(OH)s	626.0	600/599	616.9	219
δ(CC=O)1 + δ(CC=O)2	*603.0*	571	599.7	82
δ(CCO)1 + δ(CCO)2	*544.0*	*537*	540.4	43
τ(C=C)	n. i.	n. i.	148.7	<1
δ(CC=C)	n. i.	n. i.	129.6	3
τ(CC)1 + τ(CC)2	n. i.	n. i.	53.7	4

a Wavenumbers (cm^–1^); calculated intensities (km mol^–1^); s = symmetric; a = antisymmetric; ν = stretching; δ = in-plane bending; γ = out-of-plane bending; τ = torsion; n. obs. = not observed; and n. i. = not investigated. 1 and 2 refer to each one of the two equivalent-by-symmetry carboxylic groups. Frequency values shown in *italic* are assigned to more than one conformer.

**Table 7:** Experimental (in N_2_ and Ar Matrixes) and DFT(B3LYP)/6-311++G(d,p) Calculated Infrared Data for Conformer **II** of FA with the Proposed Assignments^a^

	experimental		calculated	
approximate description	N_2_ matrix	Ar matrix^32^	ν	*I*^IR^
ν(OH)1	3548.0	*3576*	3590.0	81
ν(OH)2	3541.0/3533.5	*3560*	3582.0	90
ν(CH)2 + ν(CH)1	3097.0	*2948*	3086.1	<1
ν(CH)1 + ν(CH)2	3050.5	n. obs.	3065.2	<1
ν(C=O)1	1758.5	*1765*	1763.3	219
ν(C=O)2	1750.0	*1763*/*175*9/*1756*/*1754*/*1750*	1745.5	218
ν(C=C) + δ(CCH)1 + δ(CCH)2	1656.0	1650	1662.0	29
δ(COH)2 + δ(CCH)2	1386.0	1364/1357	1322.9	92
δ(COH)1 + ν(CC)1 + ν(CO)1	1380.0	1338	1309.2	48
δ(CCH)1 + δ(CCH)2	n. obs.	1274/1269/1242	1277.7	20
δ(CCH)1 + δ(CCH)2 + δ(COH)2	n. obs.	1234	1229.5	35
δ(COH)1 + ν(CC)1 + ν(CO)1	1142.0/1140.0/1139.0	1158	1144.9	109
δ(COH)2 + ν(CO)2 + δ(CCH)2	1128.5	1120	1113.4	264
γ(CH)s	999.0/997.5/996.0	986	1004.9	46
ν(CC)2 + δ(CCH)2	n. obs.	938	928.5	20
γ(CH)a + τ(CC)2 + τ(CC)1	n. obs.	909	916.5	1
ν(CC)1 + τ(CO)1	n. obs.	896	881.8	5
γ(OH)a + γ(C=O)2	781.0/*780.5*	777	781.1	52
γ(OH)2 + γ(OH)1 + τ(CC)2	n. obs.	n. obs.	676.7	3
δ(CC=O)2 + δ(CC=O)1	n. obs.	n. obs.	659.4	10
δ(CC=O)1 + δ(COH)1 + δ(OCO)2	n. obs.	589	600.2	95
γ(OH)s	596.0	554	588.4	60
δ(CC=O)1 + δ(CCO)2	n. i.	546	546.9	18
γ(OH)a	n. i.	n. i.	544.5	91
δ(CCO)2 + δ(CCO)1	n. i.	n. i.	384.9	3
δ(C=CC)2 + δ(C=CC)1	n. i.	n. i.	258.3	1
τ(C=C) + τ(CC)2 + τ(CC)1	n. i.	n. i.	165.5	<1
τ(CC)2 + τ(CC)1	n. i.	n. i.	131.2	<1
δ(C=CC)a	n. i.	n. i.	116.8	<1
τ(CC)2 + τ(CC)1 + γ(C=O)a	n. i.	n. i.	42.0	<1

a Wavenumbers (cm^–1^); calculated intensities (km mol^–1^); s = symmetric; a = antisymmetric; ν = stretching; δ = in-plane bending; γ = out-of-plane bending; τ = torsion; n. obs. = not observed; and n. i. = not investigated. 1 and 2 refer to the carboxylic groups drawn on the left and right sides of the molecule in Figure 3, respectively. Frequency values shown in *italic* are assigned to more than one conformer.

**Table 8:** Experimental (in N_2_ and Ar Matrixes) and DFT(B3LYP)/6-311++G(d,p) Calculated Infrared Data for Conformer **III** of FA with the Proposed Assignments^a^

	experimental		calculated	
approximate description	N_2_ matrix	Ar matrix^32^	ν	*I*^IR^
ν(OH)2	*3558.0*/*3552.0*	*3576*	3581.9	172
ν(CH)2 + ν(CH)1	3100.0	*2948*	3090.1	1
ν(C=O)2	*1749.5*	*1765*/*1763*/*1759*/*1756*/*1754*/*1750*	1748.7	552
δ(COH)2 + δ(CCH)1	1382.0	1315/1311	1306.6	90
δ(CCH)1 + δ(CCH)2	n. obs.	1256	1252.7	27
δ(COH)1 + ν(CO)1	1134.0/1131.5	1132	1114.5	416
γ(CH)a	*992.0*/*990.0*/*989.0*	988	1005.7	47
γ(CH)s + τ(CC)1	n. obs.	902	887.4	16
γ(OH)s	*780.5*	*777*	780.2	25
δ(CC=O)1 + δ(CC=O)2	*603.0*	560	600.2	63
γ(OH)s	595.0	557	572.8	229
δ(CCO)1 + δ(CCO)2	*544.0*	*537*	536.1	23
τ(CC)1 + τ(CC)1 + τ(CC)2	n. i.	n. i.	138.9	<1
τ(CC)2	n. i.	n. i.	136.3	3
τ(CC)1 + τ(CC)2	n. i.	n. i.	41.6	3

a Wavenumbers (cm^–1^); calculated intensities (km mol^–1^); s = symmetric; a = antisymmetric; ν = stretching; δ = in-plane bending; γ = out-of-plane bending; τ = torsion; n. obs. = not observed; and n. i. = not investigated. 1 and 2 refer to the carboxylic groups drawn at the left and right sides of the molecule in Figure 3, respectively. Frequency values shown in *italic* are assigned to more than one conformer.

Fumaric acid has 30 fundamental vibrations. However, conformers **I** and **III** belong to the *C*_2*h*_ point group exhibiting only 15 infrared-active vibrations. The most intense experimental bands, observed at 3558.0/3552.0, 1749.5, 1122.5, and 626.0 cm^–1^, belong to conformer **I**, with the corresponding calculated frequencies being 3582.0, 1756.5, 1104.4, and 616.9 cm^–1^, respectively, assigned to the ν(OH)a, ν(C=O)a, δ(COH)a, and γ(OH)s modes, in good agreement with the experimental data. Intense bands of conformer **III** are observed at 3558.0/3552.0, 1749.5, 1134.0/1131.5, and 595.0 cm^–1^ (predicted to be at 3581.9, 1778.7, 1114.5, and 572.8 cm^–1^) and assigned to the ν(OH)2, ν(C=O)2, δ(COH)1 + ν(CO)1, and γ(OH)s vibrations. The bands at 914 (form **I**) and 1256 and 902 cm^–1^ (form **III**), reported by Maçôas et al.^32^ in an argon matrix, have no observable equivalent bands in the N_2_ matrix. The OH and C=O stretching modes of conformer **II** are observed at 3548.0, 3541.0/3533.5, 1758.5, and 1750.0 cm^–1^. Other intense bands assigned to this conformer are observed at 1386.0, 1139.0, and 1128.5 cm^–1^, which are ascribed to the modes described as δ(COH)2 + δ(CCH), δ(COH)1 + ν(CC)1 + ν(CO)1, and δ(COH)2 + ν(CO)2 + δ(CCH)2, respectively. For this conformer, the bands observed in the argon matrix at 1274, 1234, 938, 909, 896, and 589 cm^–1^ by Maçôas et al.^32^ were not observed in solid nitrogen (see Table 7).

#### 4.3.2 Conformers **IV** and **VII**: Selective NIR Irradiation at 6936.5 cm^–1^

**Scheme 3:**
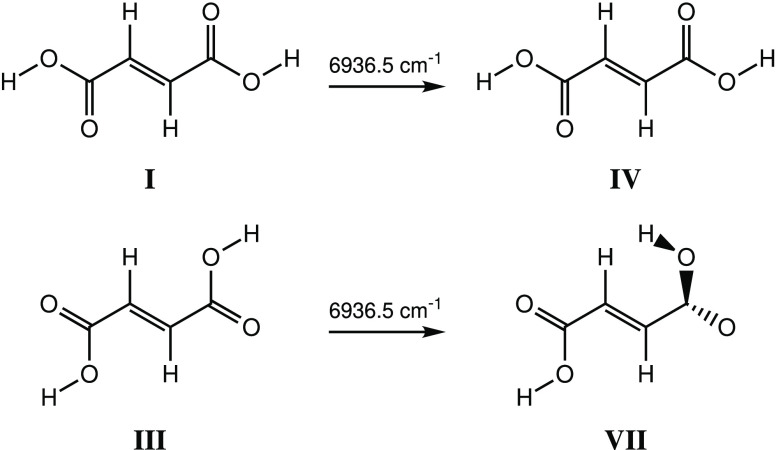
Narrowband Vibrational Excitation of the First OH Stretching Overtone at 6936.5 cm^–1^ of Conformers **I** and **III** of FA and the Formation of Conformers **IV** and **VII**

**Figure 12:**
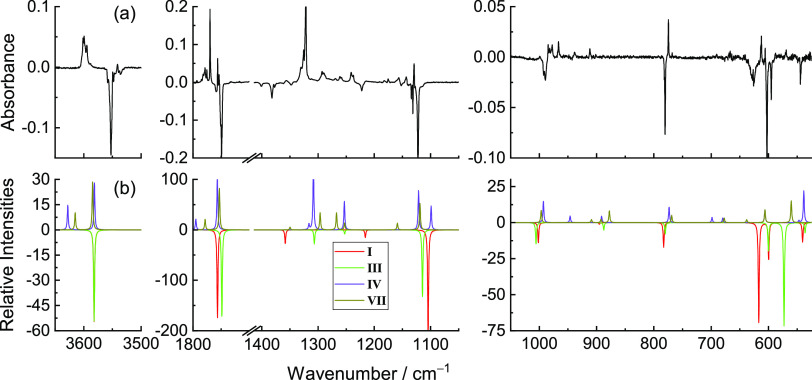
Experimental difference FTIR spectrum of FA after irradiation at 6936.5 cm^–1^ for 35 min minus the spectrum of the freshly deposited MA in a N_2_ matrix at 15 K (a). Simulated difference infrared spectra with the anharmonic frequencies of conformers **I** and **III** (pointing downward; red and green) and conformers **IV** and **VII** (pointing upward; purple and olive) (b) calculated at the DFT(B3LYP)/6-311++G(d,p) level.

Figure S2 shows a fragment of the NIR experimental spectrum of fumaric acid isolated in solid N_2_ at 15 K, together with the simulated DFT(B3LYP)/6-311++G(d,p) anharmonic spectra of conformers **I**–**III**. Excitation at 6936.5 cm^–1^, corresponding to the first overtone OH stretching vibrations whose fundamental bands are observed at 3552.0 and 3558.0 cm^–1^ and that are assigned to conformers **I** and **III**, respectively, was found to induce the formation of conformers **IV** and **VII** via light-induced rotation of the free OH groups of conformers **I** and **III**, respectively (Scheme 3, Figure 12).

The assignments of the bands that originated in photogenerated conformers **IV** and **VII** are given in Tables 9 and 10. The OH stretching bands (ν(OH)2) are observed at 3601.0/3599.5/3598.0 and 3595.0 cm^–1^ for conformers **IV** and **VII**, respectively. On the other hand, the (ν(OH)1) vibrational mode is not observed for these conformers. In the C=O stretching region, the new bands appear as a multiplet at 1785.0/1782.0/1779.0/1776.0/1770.0 cm^–1^ (**VII**, calculated frequency for ν(C=O)2, 1778.5 cm^–1^), 1793.0 cm^–1^ (**IV**, calculated frequency for ν(C=O)2 + δ(COH)2, 1795.0 cm^–1^), and 1756.0/1754.0 cm^–1^ (**IV**, calculated frequency for ν(C=O)1 + δ(COH)1, 1756.8 cm^–1^, and **VII**, calculated frequency for ν(C=O)1, 1753.1 cm^–1^). Other intense bands assigned to conformer **IV** are observed at 1325.0, 1322.0, 1241.0/1239.0/1237.0, 1142.0/1140.0, and 1129.5/1126.0/1125.0 cm^–1^ (Table 9), whereas for conformer **VII** additional bands are observed at 1330.0, 1293.0/1290.0, 1269.0/1261.0/1258.5, 1241.0/1239.0/1237.0, 1156.0, and 1139.0 cm^–1^ (Table 10).

**Table 9:** Experimental (in the N_2_ Matrix) and DFT(B3LYP)/6-311++G(d,p) Calculated Infrared Data for Conformer **IV** of FA with the Proposed Assignments^a^

	experimental	calculated	
approximate description	N_2_ matrix	ν	*I*^IR^
ν(OH)2	*3601.0*/3599.5/*3598.0*	3628.0	46
ν(OH)1	n. obs.	3581.4	88
ν(CH)1	n. obs.	3070.6	1
ν(CH)2	n. obs.	3036.4	2
ν(C=O)2 + δ(COH)2	1793.0	1795.0	67
ν(C=O)1 + δ(COH)1	*1756.0*	1756.8	328
ν(C=C) + δ(CCH)1 + δ(CCH)2	n. obs.	1666.8	1
δ(COH)1 + δ(CCH)1 + ν(CC)1 + ν(CO)1	1325.5	1316.2	36
δ(CCH)2	1322.0	1308.2	408
δ(COH)2 + ν(CO)2	*1241.0*/*1239.0*/*1237.0*	1253.3	180
δ(CCH)1 + δ(COH)1 + δ(COH)2	n. obs.	1213.1	<1
δ(COH)1 + ν(CO)1	1142.0/1140.0	1121.4	245
δ(COH)2 + δ(C=CH)2 + ν(CO)2	1129.5/1126.0/1125.0	1099.1	149
γ(CH)s	995.0/978.0/966.5	992.9	47
ν(CC)1 + ν(CC)2 + δ(CCH)1 + δ(CCH)2	940.0/938.5	946.2	15
γ(CH)a + τ(CC)2 + τ(CC)1	n. obs.	911.6	1
ν(CC)a + ν(CO)a	n. obs.	891.3	14
γ(C=O)s + γ(OH)1	*775.0*	773.4	34
δ(OCO)a	n. obs.	698.6	11
γ(OH)1 + τ(CC)2 + τ(CC)1	*666.5*	679.8	10
δ(CC=O)1 + δ(CC=O)2	*613.5*/*612.0*/*606.0*	606.2	28
γ(OH)1 + γ(C=O)1	n. obs.	546.8	5
δ(CCO)2 + δ(CCO)1	n. i.	538.3	71
γ(OH)2	n. i.	477.4	115
δ(CCO)1 + δ(CCO)2	n. i.	368.8	12
δ(C=CC)2 + δ(C=CC)1	n. i.	269.8	1
τ(C=C) + τ(CC)2	n. i.	147.2	6
δ(C=CC)a	n. i.	137.8	7
τ(CC)2 + τ(CC)1	n. i.	127.7	10
τ(CC)2 + τ(CC)1 + γ(C=O)a	n. i.	54.2	4

a Wavenumbers (cm^–1^); calculated intensities (km mol^–1^); s = symmetric; a = antisymmetric; ν = stretching; δ = in-plane bending; γ = out-of-plane bending; τ = torsion; n. obs. = not observed; and n. i. = not investigated. 1 and 2 refer to the *cis* and *trans* carboxylic groups, respectively. Frequency values shown in *italic* are assigned to more than one conformer.

**Table 10:** Experimental (in the N_2_ Matrix) and DFT(B3LYP)/6-311++G(d,p) Calculated Infrared Data for Conformer **VII** of FA with the Proposed Assignments^a^

	experimental	calculated	
approximate description	N_2_ matrix	ν	*I*^IR^
ν(OH)2	3595.0	3615.0	32
ν(OH)1	n. obs.	3584.7	92
ν(CH)2	n. obs.	3076.2	<1
ν(CH)1	n. obs.	3043.3	4
ν(C=O)2	1785.0/*1782.0*/*1779.0*/*1776.0*/*1770.0*	1778.5	66
ν(C=O)1	*1754.0*	1753.1	262
ν(C=C) + δ(CCH)1 + δ(CCH)2	n. obs.	1648.0	1
δ(COH)1 + ν(CO)1	1330.0	1349.8	17
δ(CCH)1	1293.0/1290.0	1296.3	108
δ(COH)2 + ν(CO)2	1269.0/1261.0/1258.5	1267.2	109
δ(CCH)1 + δ(CCH)2	*1241.0*/*1239.0*/*1237.0*	1252.8	43
δ(COH)1 + δ(COH)2	1156.0	1159.0	41
δ(COH)1 + δ(COH)2	1139.0	1118.9	168
γ(CH)s	999.0/998.0/996.0	996.7	27
γ(CH)a + τ(CC)1 + τ(CC)2	912.0	908.9	7
τ(CC)1 + τ(C=C) + ν(CO)1	n. obs.	892.4	7
τ(CC)2 + τ(C=C) + ν(CO)2	n. obs.	877.7	26
γ(OH)1 + γ(C=O)s	n. obs.	769.0	16
γ(C=O)a	*666.5*	677.3	10
γ(OH)s	644.0	638.0	7
δ(OCO)1 + δ(OCO)2	*613.5*/*612.0*/*606.0*	606.1	28
γ(OH)1	570.0	560.2	49
γ(OH)a	520.0	514.1	85
γ(OH)2	n. i.	439.5	28
δ(CCO)1 + δ(CCO)2	n. i.	391.7	32
τ(CO)2	n. i.	247.7	2
τ(C=C)	n. i.	143.1	7
τ(CC)1 + τ(CC)2	n. i.	156.5	13
τ(CC)1	n. i.	99.8	1
τ(CC)1 + τ(CC)2	n. i.	39.9	10

a Wavenumbers (cm^–1^); calculated intensities (km mol^–1^); s = symmetric; a = antisymmetric; ν = stretching; δ = in-plane bending; γ = out-of-plane bending; τ = torsion; n. obs. = not observed; and n. i. = not investigated. 1 and 2 refer to the *cis* and *trans* carboxylic groups, respectively. Frequency values shown in *italic* are assigned to more than one conformer.

#### 4.3.3 Conformers **V** and **VI**: Selective NIR Irradiation at 6909.5 cm^–1^

**Scheme 4:**
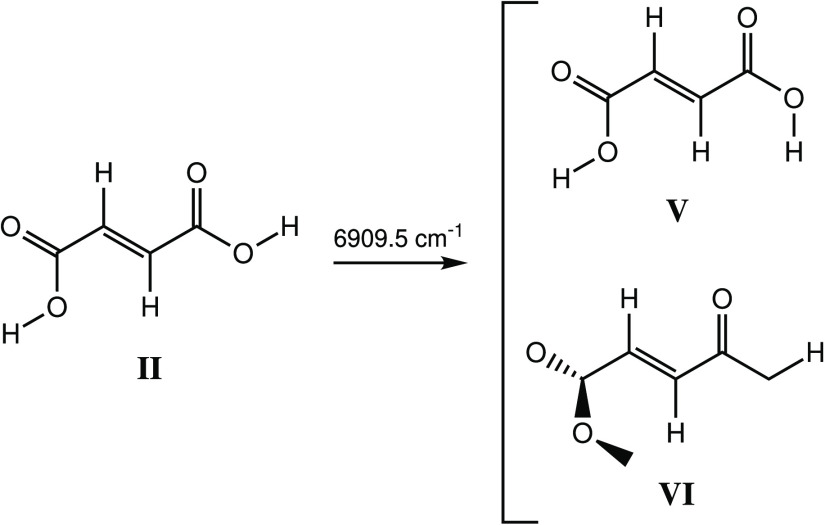
Narrowband Vibrational Excitation of the First OH Stretching Overtone at 6909.5 cm^–1^ of Conformer **II** of FA and the Formation of Conformers **V** and **VI**

**Figure 13:**
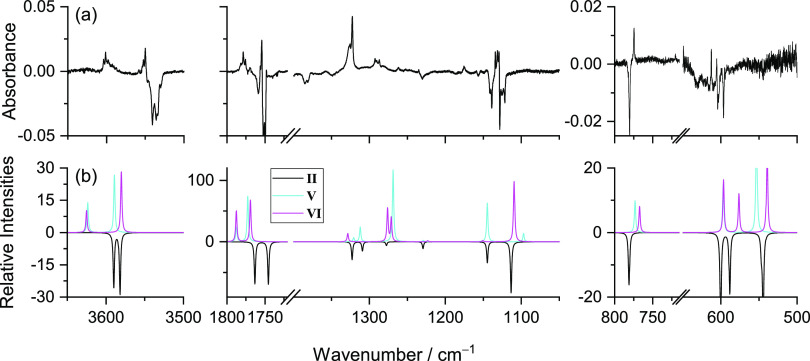
Experimental difference FTIR spectrum of FA after irradiation at 6909.5 cm^–1^ for 3 h 30 min minus the spectrum of the freshly deposited FA in a N_2_ matrix at 15 K (a). Simulated difference infrared spectra with the anharmonic frequencies of conformer **II** (pointing downward; black) and conformers **V** and **VI** (pointing upward; blue and pink) (b) calculated at the DFT(B3LYP)/6-311++G(d,p) level.

Irradiation at 6909.5 cm^–1^ corresponds to the excitation of the first overtone of the OH stretching vibrations of conformer **II** (fundamental bands: 3548.0 and 3541.0/3533.5 cm^–1^; see Table 7). Upon this excitation, conformer **II** was found to convert into conformers **V** and **VI**, which differ from conformer **II** only by the rotation of one of the OH groups (Scheme 4, Figure 13).

Tables 11 and 12 list the assignments of the fundamental bands for conformers **V** and **VI**, compared with the corresponding calculated spectra. Bands due to conformer **V** are observed at 3604.0/3601.0, 1782.0/1779.0/1776.0/1770.0, 1649.5, 1326.0, 1322.5, 1287.0, 1134.0/1131.5, 983.0, 912.0, 775.0, and 612.5 cm^–1^ (see Table 11 for details), whereas those ascribed to conformer **VI** are observed at 3598.0, 1782.0/1779.0/1776.0/1770.0, 1293.0, 1290.5/1287.0, 1129.0, 979.0, 943.5, and 612.5 cm^–1^ (Table 12).

**Table 11:** Experimental (in the N_2_ Matrix) and DFT(B3LYP)/6-311++G(d,p) Calculated Infrared Data for Conformer **V** of FA with the Proposed Assignments^a^

	experimental	calculated	
approximate description	N_2_ matrix	ν	*I*^IR^
ν(OH)2	3604.0/*3601.0*	3623.5	45
ν(OH)1	n. obs.	3589.2	86
ν(CH)1	n. obs.	3073.2	1
ν(CH)2	n. obs.	3042.6	3
ν(C=O)2	*1782.0*/*1779.0*/*1776.0*/1770.0	1787.5	74
ν(C=O)1	n. obs.	1772.8	238
ν(C=C) + δ(CCH)1 + δ(CCH)2ν	1649.5	1656.8	29
δ(COH)1 + ν(CC)1 + ν(CO)1	1326.0	1312.2	76
δ(CCH)2	1322.5	1320.9	21
δ(CCH)1 + δ(COH)2 + ν(CO)2	*1287.0*	1268.9	370
δ(CCH)1 + δ(CCH)2 + δ(COH)2	n. obs.	1229.1	7
δ(COH)1 + ν(CC)1 + ν(CO)1	1134.0/1131.5	1144.9	200
δ(COH)2 + δ(CCH)2 + ν(CO)2	n. obs.	1097.2	44
γ(CH)s	983.0	1000.2	46
ν(CC)2 + δ(CCH)2	912.0	921.6	44
γ(CH)a	n. obs.	909.0	2
ν(CC)1 + ν(CO)1	n. obs.	878.0	4
γ(C=O)s + γ(OH)a	*775.0*	773.3	31
δ(CC=O)2	n. obs.	675.1	1
γ(OH)s	n. obs.	661.4	2
δ(CC=O)1 + δ(OCO)2	*612.5*	596.8	48
γ(OH)1	n. obs.	553.3	99
δ(CC=O)1 + δ(CC=O)2	n. obs.	551.9	11
γ(OH)2	n. i.	455.1	47
δ(CCO)2 + δ(CCO)1	n. i.	386.2	8
δ(C=CC)2 + δ(C=CC)1	n. i.	264.1	3
τ(C=C) + τ(CC)2 + τ(CC)1	n. i.	152.0	4
δ(C=CC)	n. i.	134.0	4
τ(C=C)	n. i.	128.0	3
τ(CC)1 + τ(CC)2	n. i.	49.4	<1

a Wavenumbers (cm^–1^); calculated intensities (km mol^–1^); s = symmetric; a = antisymmetric; ν = stretching; δ = in-plane bending; γ = out-of-plane bending; τ = torsion; n. obs. = not observed; and n. i. = not investigated. 1 and 2 refer to the *cis* and *trans* carboxylic groups, respectively. Frequency values shown in *italic* are assigned to more than one conformer.

**Table 12:** Experimental (in the N_2_ Matrix) and DFT(B3LYP)/6-311++G(d,p) Calculated Infrared Data for Conformer **VI** of FA with the Proposed Assignments^a^

	experimental	calculated	
approximate description	N_2_ matrix	ν	*I*^IR^
ν(OH)1	*3598.0*	3625.1	33
ν(OH)2	n. obs.	3580.3	91
ν(CH)1	n. obs.	3063.5	<1
ν(CH)2	n. obs.	3039.5	3
ν(C=O)1	*1782.0*/*1779.0*/*1776.0*/*1770.0*	1787.6	162
ν(C=O)2	n. obs.	1769.2	219
ν(C=C) + δ(CCH)2	n. obs.	1651.6	18
δ(COH)2 + δ(CCH)2	n. obs.	1328.7	43
δ(COH)1 + ν(CC)1 + ν(CO)1	1293.0	1276.2	174
δ(CCH)2	1290.5/*1287.0*	1271.3	123
δ(CCH)1 + δ(CCH)2 + δ(COH)2	n. obs.	1223.3	6
δ(COH)2 + ν(CC)1	n. obs.	1150.9	6
δ(COH)2 + ν(CO)2	1129.0	1109.5	312
γ(CH)s	979.0	997.8	38
ν(CC)2	943.5	937.7	14
γ(CH)a	n. obs.	900.7	2
ν(CC)1 + ν(CO)1	n. obs.	873.9	24
γ(C=O)s + γ(OH)a	n. obs.	767.3	26
δ(CC=O)2 + δ(OCO)2	n. obs.	681.1	18
γ(OH)2	n. obs.	665.3	5
δ(CC=O)1 + δ(CC=O)2	*612.5*	596.4	52
τ(CO)2	n. i.	576.3	39
τ(CO)1	n. i.	539.3	80
γ(OH)1	n. i.	426.8	38
δ(CCO)2 + δ(CCO)1	n. i.	381.7	36
δ(C=CC)	n. i.	253.4	2
τ(CO)2	n. i.	158.9	4
τ(CC)1 + τ(CC)2	n. i.	114.9	8
τ(C=C)	n. i.	126.4	6
τ(CC)1 + τ(CC)2	n. i.	40.4	2

a Wavenumbers (cm^–1^); calculated intensities (km mol^–1^); s = symmetric; a = antisymmetric; ν = stretching; δ = in-plane bending; γ = out-of-plane bending; τ = torsion; n. obs. = not observed; anb n. i. = not investigated. 1 and 2 refer to the *trans* and *cis* carboxylic groups, respectively. Frequency values shown in *italic* are assigned to more than one conformer.

Note that the irradiation at 6936.5 cm^–1^ (of **I** and **III**, described in the previous section) and 6909.5 cm^–1^ (**II**) produces some common bands which can be assigned simultaneously to different conformers. However, the efficiency of the consumption of the reactants is quite different and therefore allows discrimination of the different processes (see Figure S3 in the Supporting Information).

#### 4.3.4 Tunneling Reactions: **IV** → **I**, **VII**→ **III**, and **V/VI**→ **II** Decays in the Dark

**Figure 14:**
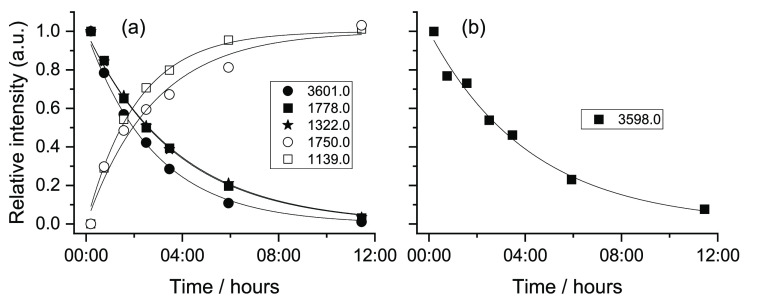
Tunneling decay of FA conformers **V** (a) and **VI** (b) in a N_2_ matrix at 15 K after irradiation at 6909.5 cm^–1^. The decays were evaluated using bands at 3601.0 (ν(OH)2), 1778.0 (ν(C=O)2), and 1322.0 cm^–1^ (δ(CCH)2) (**V**) and at 3598.0 cm^–1^ ((ν(OH)1) (**VI**). The recovery of conformer **II** was evaluated using the bands at 1750.0 (ν(OH)2) and 1139.0 cm^–1^ (δ(COH)1 + ν(CC)1 + ν(CO)1). The results were fitted with single-exponential functions.

**Figure 15:**
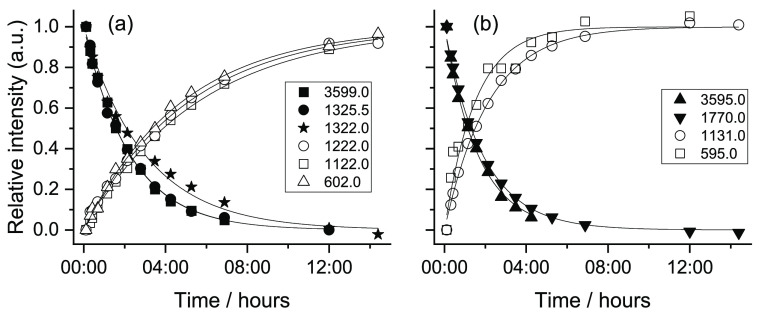
Tunneling decays of FA conformers **IV** (a) and **VII** (b) in a N_2_ matrix at 15 K after irradiation at 6936.5 cm^–1^. The decay of form **IV** was evaluated using bands at 3599.0 (ν(OH)2), 1325.5 (δ(COH)1 + δ(CCH)1 + ν(CC)1 + ν(CO)1), and 1322.0 cm^–1^ (δ(CCH)2); the decay of form **VII** was evaluated using bands at 3599.0 (ν(OH)2) and 1770.0 cm^–1^ (ν(C=O)2). The recovery of conformer **I** (a) was evaluated using the bands at 1222.0 (δ(C=CH)a + δ(COH)a), 1122.0 (δ(COH)a), and 602 cm^–1^ (δ(CC=O)1 + δ(CC=O)2). The recovery of conformer **III** (b) was evaluated using the bands at 1131.0 (δ(COH)1 + ν(CO)1) and 595.0 cm^–1^ (γ(OH)s). The results were fitted with single-exponential functions.

As for maleic acid, the tunneling decay processes of the new forms of FA generated as a result of the performed NIR irradiation were also investigated. Figure 14 shows the tunneling decays of conformers **V** and **VI** (to conformer **II**), following their production by irradiation at 6909.5 cm^–1^. The observed lifetimes (average values) were found to be ca. 2 h 40 min (**V**) and 3 h (**VI**). On the other hand, the tunneling decays of conformers **IV** and **VII** (into forms **I** and **III**, respectively), following their production by irradiation at 6936.5 cm^–1^, are shown in Figure 15. The lifetime of conformer **IV** is about 1 h 30 min, while conformer **VII** was found to have a lifetime of *ca*. 1 h 15 min.

It shall be noticed that in some cases the recovered bands of forms **I**–**III** are slightly shifted in relation to their position in the spectra of the as-deposited matrixes, in view of the possible differences in the sites where the molecules stand.

The comparison of the relative stabilities of the photogenerated *cis–trans* conformers of FA (**IV**–**VII**) in the N_2_ matrix and also with the photogenerated conformers of maleic acid (**VI** and **VII**) reveals that FA conformers **V** and **VII** follow the expected pattern, with their lifetimes correlating well with their relative energies and the barriers for their decay into FA conformers **II** and **III**, respectively (the barriers are 25.2 and 22.4 kJ mol^–1^ and the corresponding lifetimes are ca. 2 h 40 min and 1 h 15 min, respectively; the barriers and lifetimes for conformers **VI** and **VII** of MA are 23.7 and 27.8 kJ mol^–1^ and ca. 2 and 5 h 30 min, respectively). On the other hand, taking into account the height of the barriers for their isomerization to conformers **II** and **III**, respectively 19.0 and 30.2 kJ mol^–1^, FA conformer **VI** would be expected to be less stable than experimentally observed and conformer **VII** would be expected to be more stable. However, it is well known that the relative rates of tunneling decay of carboxylic acid conformers in low-temperature matrixes do not depend only on the intrinsic profile of their barriers for conformational isomerization [though this is usually the most relevant factor, in the case of N_2_ matrixes together with the specific H-bond-like stabilizing interaction between the free OH moiety(ies) present in the molecule and the N_2_ matrix molecules, as already pointed out but otherwise resulting from a combination of different factors (e.g., the efficiency of solvation, matrix sites, and the mismatch between the tunneling levels in the reactant and product species)^58^ whose relative importance is sometimes impossible to estimate].

### 4.4 Broadband UV Irradiation (λ > 235 nm)

**Scheme 5:**
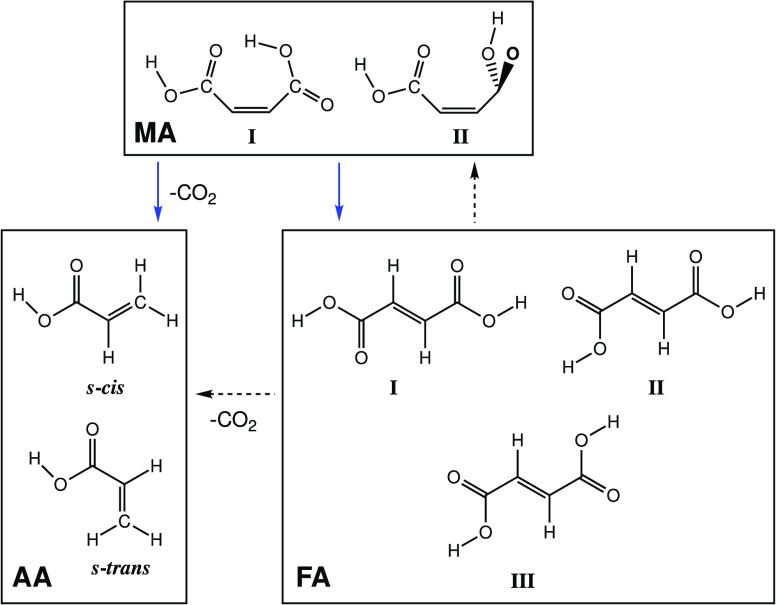
Broadband UV Irradiation (λ > 235 nm) of Maleic (MA) and Fumaric (FA) Acids and the Formation of Acrylic Acid (AA) Solid blue arrows show the products of irradiation of MA, and dashed black arrows show the products of irradiation of **FA**.

The UV-induced photochemistries of maleic and fumaric acids isolated in solid N_2_ were also investigated in the present study. After irradiation of the MA with broadband (λ > 235 nm) UV light, provided by a high-pressure Hg/Xe arc lamp fitted with a water filter, bands belonging to the initial compound decreased in intensity while new bands emerged. Changes were observed immediately after 1 min of irradiation time (the total time was 70 min). Scheme 5 shows the reaction mechanisms considered for the isomerization of maleic acid into fumaric acid and vice versa and the formation of acrylic acid with the release of carbon dioxide (CO_2_) resulting from decarboxylation from both acids.

For maleic acid, two main photoreactions were observed: isomerization of maleic acid to fumaric acid and decarboxylation leading to the formation of acrylic acid (CH_2_=CHCOOH), which is produced in two different conformers (see Scheme 5 and Figure S4 in the Supporting Information).^59,60^ The new bands observed at 3552.0, 3548.0, 1750.5, 1748.0, 1134.0/1131.5, 1122.5, 999.0, 996.0, 992.0, 990.0, 782.0/780.5, 602.0, and 596.0 cm^–1^ are due to fumaric acid, whereas bands due to the *s-trans* conformer of acrylic acid are observed at 3600.0/3588.0 cm^–1^ (ν(OH), predicted at 3588.4 cm^–1^), 1760.0/1759.0 cm^–1^ (ν(C=O), predicted at 1759.8 cm^–1^), 1307.5/1304.0 cm^–1^ (δ(COH) + ν(CO)), predicted at 1307.4 cm^–1^), 1026.0/1024.5 and 1021.0 cm^–1^ (w(CH_2_) and τ(C=C), predicted respectively at 1014.9 and 1007.3 cm^–1^), 992.0 and 990.0 cm^–1^ (γ(CH_2_), theory: 990.1 cm^–1^), 819.0/817.0 cm^–1^ (ν(CC) and γ(C=O), theory: 822.0 and 815.8 cm^–1^), and 585.5 cm^–1^ (δ(O=CO), predicted at 580.4 cm^–1^) (see Table 13). Additional bands were also detected in the spectra of the irradiated matrix that could be assigned to the *s-cis* conformer of acrylic acid (see Table S5 in the Supporting Information). These bands appear at 1122.0 (δ(COH), theory: 1119.9 cm^–1^), 999.0/996.0 (w(CH_2_), theory: 999.0 cm^–1^), and 616.0 cm^–1^ (γ(OH), theory: 613.2 cm^–1^).

**Table 13:** Experimental (in N_2_ and Ar Matrices) and DFT(B3LYP)/6-311++G(d,p) Calculated Infrared Data (Anharmonic) for the *s-trans* Conformer of Acrylic Acid, with the Proposed Assignments^a^

	experimental		calculated	
approximate description	N_2_ matrix	Ar matrix^59^	ν	*I*^IR^
ν(OH)	3600.0/3588.0	3575.9/3571.5	3588.4	62.3
ν(CH_2_)as	n. obs.	n. obs.	3102.3	2.4
ν(CH)	n. obs.	n. obs.	3058.5	2.2
ν(CH_2_)s	n. obs.	n. obs.	2992.8	3.1
ν(C=O)	1760.0/1759.0	1759.9/1757.4/1755.9	1759.8	366.1
ν(C=C) + δ(CH_2_)	n. obs.	1651.4/1625.7/1623.8	1649.5	9.5
δ(CH_2_)	n. obs.	1416.0	1411.1	22.9
δ(COH) + ν(CO)	1307.5/1304.0	1329.7	1307.4	46.5
δ(CH) + w(CH_2_)	n. obs.	n. obs.	1283.7	2.3
δ(COH)	1175.0	1187.0/1184.6	1183.1	135.5
w(CH_2_)	1026.0/1024.5	1019.2/1016.9	1014.9	109.7
τ(C=C)	1021.0	998.6/991.2	1007.3	14.0
γ(CH_2_)	992.0/990.0	970.6/968.1/967.1	990.1	43.7
ν(CC)	819.0	825.9	822.0	34.2
γ(C=O)	817.0	816.2	815.8	6.0
δ(O=CO)	585.5	580.5	580.4	94.4
τ(CO)	n. obs.	568.4/567.0	578.5	33.3
δ(CC=O)	n. obs.	525.0	523.3	3.5
γ(C=O) + γ(CH)	n. i.	475.9/472.0	473.3	17.6
δ(C=CC) + δ(CC=O)	n. i.	n. i.	282.9	1.0
τ(CC)	n. i.	n. i.	112.4	0.0

a Wavenumbers (cm^–1^); calculated intensities (km mol^–1^); s = symmetric; a = antisymmetric; ν = stretching; δ = in-plane bending; γ = out-of-plane bending; τ = torsion; n. obs. = not observed; and n. i. = not investigated.

Similar results were obtained after the UV irradiation (λ > 235 nm) of FA in a N_2_ matrix. However, for this molecule, the efficiency was much higher because the initial reactant was almost completely consumed (∼94%) after irradiation for a total time of only 35 min (see Figure S5 in the Supporting Information). It is clear from the spectroscopic data that the main photoreaction observed was the isomerization of fumaric acid to maleic acid, with the new bands observed at 3534.0, 3527.0/3521.0, 1770.0, 1759.0, 1726.5/1722.0/1718.0, 1640.0, 1444.0/1433.0, 1418.0, 1168.0/1165.0/1157.5, 939.0, 863.0, 846.0, 839.0, 833.5, 787.0, 561.0, and 559.0 cm^–1^, ascribable to conformers **I** and **II** of maleic acid. The decarboxylation of FA, to give acrylic acid, was also observed as a minor photoreaction. Bands assigned to the *s-trans* form of acrylic acid were observed at 3588.0, 1760.0/1759.0, 1307.5/1304.0, 1175.0 (δ(COH), theory: 1183.1 cm^–1^), and 819.0 cm^–1^, whereas bands at 1779.0 (ν(C=O), predicted at 1764.5 cm^–1^), 1652.0 (ν(C=C) + δ(CH_2_), predicted at 1661.8 cm^–1^), 1396.0 (δ(CH_2_) + δ(CH), theory: 1405.1 cm^–1^), 827.0 (ν(CC), theory: 818.6 cm^–1^), 615.0 (τ(CO), 621.6 cm^–1^), and 611.0 cm^–1^ (δ(O=CO) + δ(CC=O), 613.3 cm^–1^) were assigned to the *s-cis* conformer of acrylic acid.

**Figure 16:**
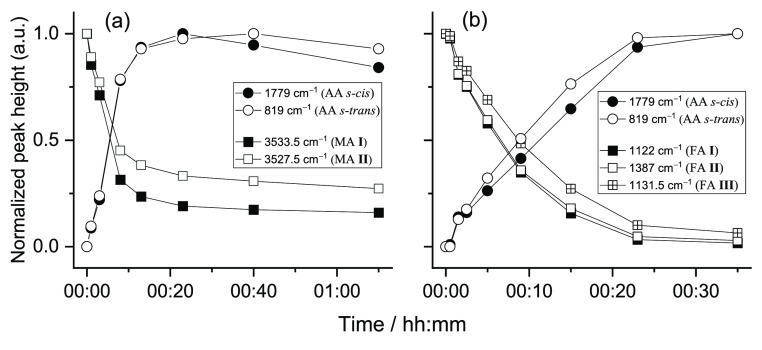
Kinetics of characteristic peaks of maleic (MA), fumaric (FA), and acrylic acids (AA) as a function of the time of UV irradiation (λ > 235 nm) of MA (a) and FA (b) in a N_2_ matrix.

To ascertain the kinetics of the dissociation of maleic and fumaric acids and the formation of acrylic acid, the intensities of the corresponding characteristic peaks of the acids’ conformers were plotted as a function of total UV-irradiation time (Figure 16). On the basis of the obtained results, the dissociation rates of conformers **I** and **II** of maleic acid (Figure 16(a)) and those of the three conformers of fumaric acid (Figure 16(a)) appear to be almost the same. A similar conclusion can be drawn about the formation rates of the two conformers of the acrylic acid (Figure 16(a,b)).

It shall be highlighted that no conformational interconversions between the observed conformers of each of the acids studied could be convincingly detected to take place upon UV irradiation. In the work by Maçôas et al.,^32^ the UV irradiation (λ = 266 nm) of fumaric acid in an argon matrix was found to efficiently promote the isomerization of fumaric acid into maleic acid, as also observed now in the N_2_ matrix. However, the decarboxylation of FA was not reported in the argon matrix study, and no data was presented regarding the behavior of maleic acid upon UV irradiation.

## 5 Conclusions

In this study, monomers of maleic and fumaric acids were investigated with IR spectroscopy in a low-temperature (15 K) nitrogen matrix. The data analysis was supported by electronic structure and vibrational calculations performed at the DFT(B3LYP) and MP2 levels of theory.

The two most stable conformers of maleic acid (**I** and **II**) and the three most stable forms of fumaric acid (**I**–**III**) were identified in the as-deposited matrixes. Selective excitation using narrowband NIR radiation tuned at the first overtone OH stretching vibrations of these conformers allowed their conversion to novel high-energy conformers of the two molecules. For maleic acid, conformers **VI** and **VII** were produced by vibrational excitation (irradiation at 6898.5, 6883.5, and 6874.0 cm^–1^) of their structurally related conformers **I** and **II**. Besides, the NIR-induced conversion of conformer **II** to conformer **I** was also observed upon irradiation at 6874.0 cm^–1^. In the case of fumaric acid, the conversion of forms **I** and **III** into conformers **IV** and **VII**, respectively, after irradiation at 6936.5 cm^–1^, and of conformer **II** into forms **V** and **VI**, after irradiation at 6909.5 cm^–1^, was observed.

Once formed, the new high-energy forms decay via a hydrogen atom tunneling mechanism. The lifetimes of the photogenerated conformers in a N_2_ matrix were obtained, being 5 h 30 min (**VI**) and 2 h (**VII**) in the case of the conformers of maleic acid and 1 h 30 min (**IV**), 2 h 40 min (**V**), 3 h (**VI**), and 1 h 15 min (**VII**) for the conformers of fumaric acid. With the exception of conformers **VI** and **VII** of fumaric acid, the lifetimes of the high-energy conformers of the two molecules correlate well with the energy barriers for their conversion to the most stable conformers to which they decay.

Irradiation of matrix-isolated MA and FA with broadband UV light (λ > 235 nm) was found to result in their isomerization to FA and MA, respectively, as the major reaction pathway and also in decarboxylation to acrylic acid.

The performed theoretical analysis of the potential energy surfaces of the two molecules expands the previously available data for the two molecules, whereas the observation of all high-energy conformers generated upon NIR vibrational excitation of their most stable precursor forms allowed the characterization of six new forms of the compounds for the first time. Besides, the UV-induced photochemistry of the two studied acids in the N_2_ matrix has been now reported, with the identification of two reaction channels: *cis–trans* (maleic–fumaric) photoisomerization (major path) and decarboxylation.

## Supporting Information Available

Tables S1–S4, with DFT(B3LYP)/6-31++G(d,p) calculated optimized bond lengths and angles for the conformers of maleic and fumaric acids and their calculated anharmonic IR spectra; Table S5, with the assignments of IR bands for conformer *s-cis* of acrylic acid; Figures S1 and S2, showing fragments of the NIR experimental spectrum of maleic acid and fumaric acids in a N_2_ matrix, compared with the simulated anharmonic spectra for the relevant conformers; Figure S3, showing the comparison of the results of the irradiation of fumaric acid in a N_2_ matrix at 6909.5 and 6936.5 cm^–1^; Figures S4 and S5, with the results of broadband UV irradiation of maleic and fumaric acids isolated in a N_2_ matrix (PDF)
