# Correction to “Matrix Isolation Study of Fumaric and Maleic Acids in Solid Nitrogen”

**DOI:** 10.1021/acs.jpca.2c08698

**Published:** 2022-12-22

**Authors:** Timur Nikitin, Susy Lopes, Rui Fausto

These corrections do not alter any conclusions of the article. The authors apologize for the errors.

i) As described in the original paper, the observed conformational isomerizations of the *C*_2*h*_ symmetry conformers **I** and **III** of fumaric acid into conformers **IV** and **VII**, respectively, occur through excitation of the OH stretching coordinates of the reactant conformers, following an identical mechanism as for maleic acid and also as for the previously studied related oxalic acid molecule.^1^ After excitation, intramolecular vibrational energy relaxation allows for energy to be deposited into the reactive torsional C–O modes, ultimately leading to the observed conformational conversions. In the case of the **I** → **IV** and **III** → **VII** conformational conversions observed for fumaric acid, the energy has been initially provided to the OH stretching coordinates of the reactant conformers via their infrared active B_u_ symmetry ν(OH)_a_ + ν(OH)_s_ combination mode, observed at 6936.5 cm^–1^ for both **I** and **III**, which have been misassigned in the original paper to the corresponding 2ν(OH)_a_ overtone mode (A_g_; infrared inactive).
  The following sentences replace the original ones (changes in *italic*):
  Abstract: “Selective narrowband near-infrared (NIR) excitation of the first OH stretching overtone *(2ν(OH)*_*a*_*) or ν(OH)*_*a*_*+ν(OH)*_*s*_*combination tone of* the different conformers of maleic and fumaric acids initially present in the matrixes allowed the generation of higher-energy forms, never before observed experimentally.”
  Page 4406: “Excitation at 6936.5 cm^–1^, corresponding *to the ν(OH)*_*a*_*+ν(OH)*_*s*_*combination mode in conformers***I***and***III**, was found to induce the formation of conformers **IV** and **VII** via light-induced rotation of the free OH groups of conformers **I** and **III**, respectively (Scheme 3, Figure 12).”
  Page 4410: “Selective excitation using narrowband NIR radiation tuned at the first OH stretching overtone *or appropriate OH combination tone* of these conformers allowed their conversion to novel high-energy conformers of the two molecules.”
ii) The symmetry of conformer **X** of fumaric acid is in fact *C*_*i*_, not *C*_1_. Corrections apply to Table 1 (last line), and page 4399 (changes in *italic*): “These conformers can be obtained from conformers **I**–**III**, respectively, by internal rotation around both C–O bonds and are of *C*_2*h*_ (**VIII**), *C*_1_*(***IX***) and C*_*i*_*(***X***)* symmetry.”
iii) The Table of Contents Graphic and Schemes 3 and 4 have errors and are reproduced here in their correct form.
iv) The titles of the ordinate scale of Figures 1 and 4 have a misspelling. The corrected figures are provided here.

**Scheme 3:**
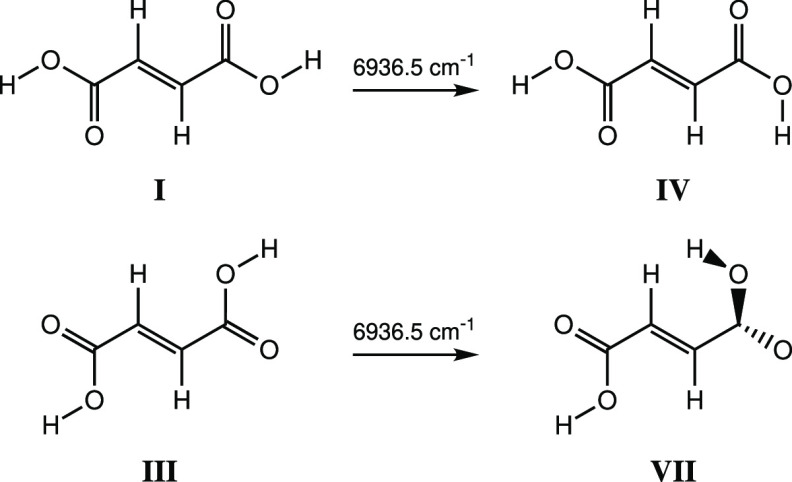
Narrowband Vibrational Excitation of the OH Stretchings’ Combination Tone at 6936.5 cm^–1^ of Conformers **I** and **III** of FA and the Formation of Conformers **IV** and **VII**

**Scheme 4:**
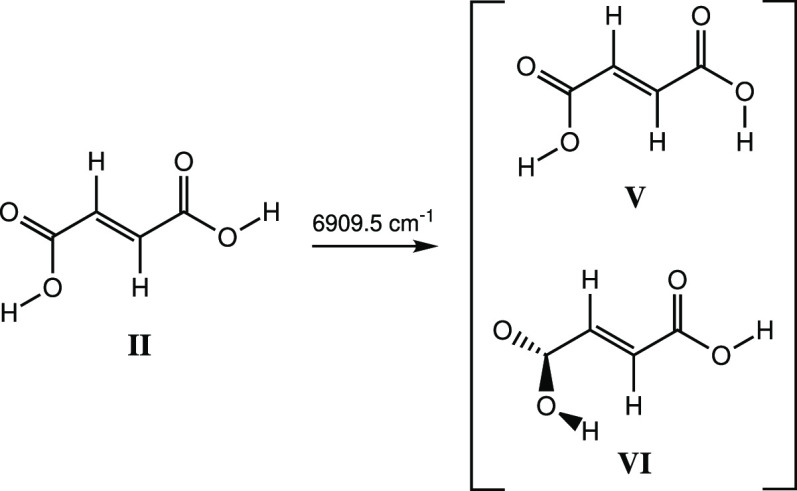
Narrowband Vibrational Excitation of the First OH Stretching Overtone at 6909.5 cm^–1^ of Conformer **II** of FA and the Formation of Conformers **V** and **VI**

**Figure 1:**
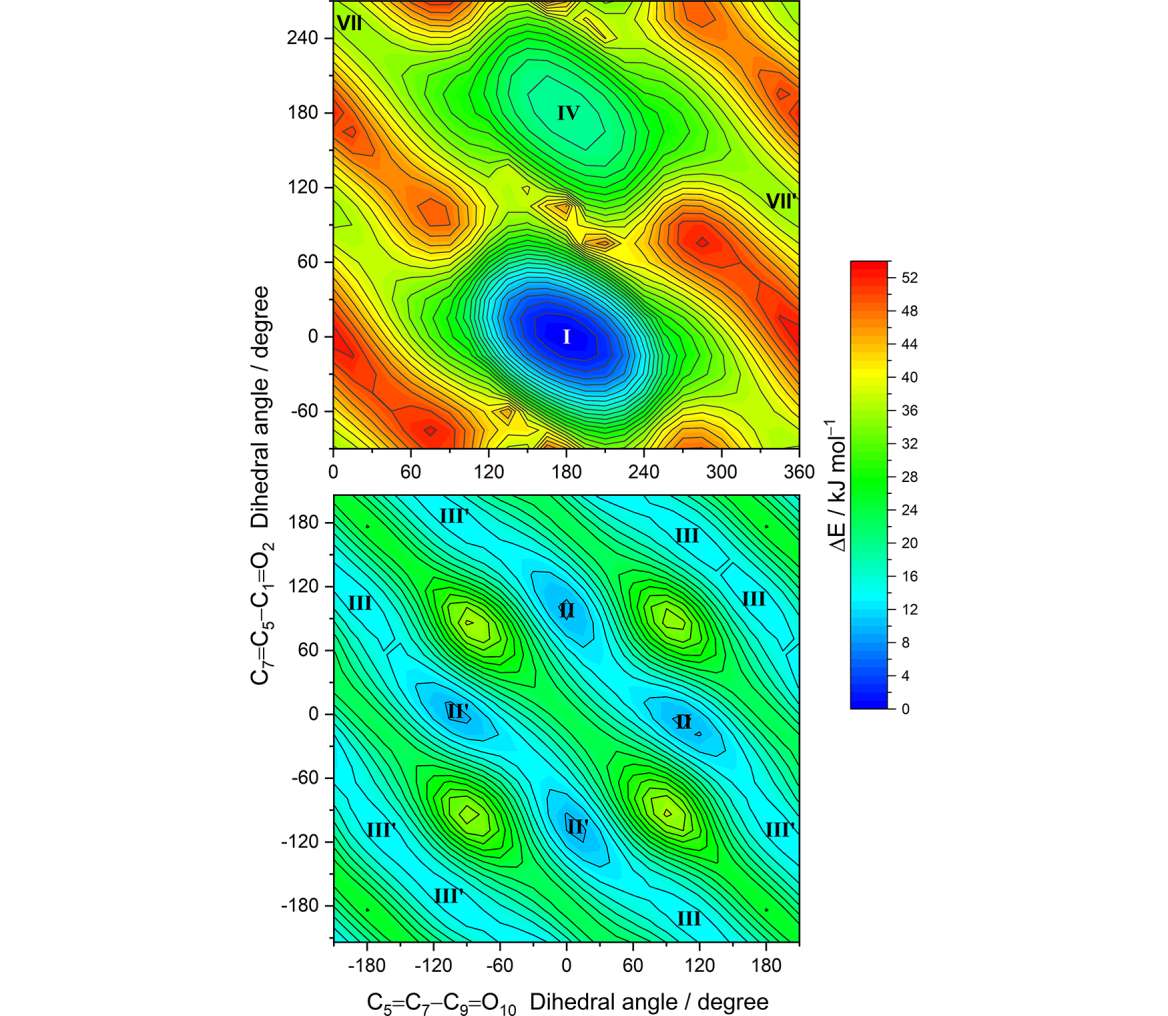
Relaxed potential energy surface contour maps of MA calculated at the DFT(B3LYP)/6-311++G(d,p) level. The C5=C7—C9=O10 and C7=C5—C1=O2 dihedral angles were incremented in steps of 15°, and all remaining internal coordinates were optimized at each point. The locations of conformers are indicated by the Roman numerals. The color bar designates the energy scale defined relative to the electronic energy of the lowest-energy form **I** (without the zero-point vibrational corrections). The isoenergy contour lines are traced using steps of 2 kJ mol^–1^.

**Figure 4:**
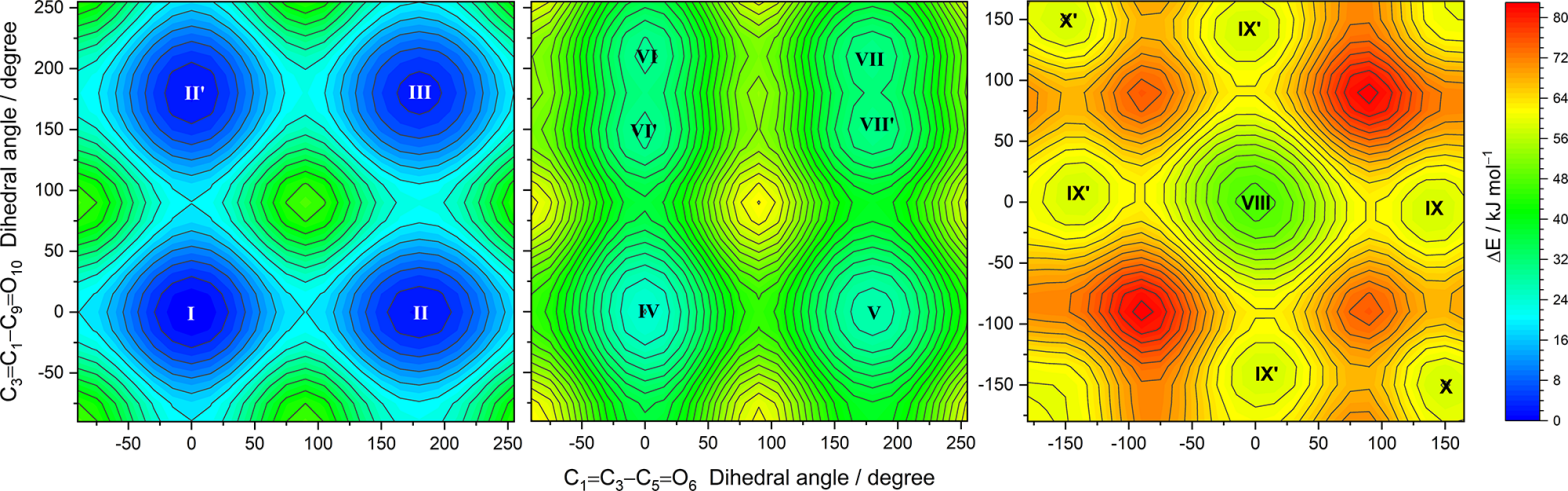
Relaxed potential energy surface contour maps of FA calculated at the DFT(B3LYP)/6-311++G(d,p) level for *cis–cis* (left panel), *cis–trans* (middle), and *trans–trans* (right) conformers. The C1=C3—C5=O6 and C3=C1—C9=O10 dihedral angles were incremented in steps of 15°, and all remaining internal coordinates were optimized at each point. The locations of six conformers are indicated by the Roman numerals. The color bar designates the energy scale defined relative to the electronic energy of the lowest-energy form **I** (without the zero-point vibrational corrections). The isoenergy contour lines are traced using steps of 2 and 4 kJ mol^–1^.


