# Supplementary material for: Matrix Isolation Study of Fumaric and Maleic Acids in Solid Nitrogen
Source: J Phys Chem A. 2022 Jun 23;126(27):4392–412. doi: 10.1021/acs.jpca.2c02770 (PMC9776572; doi:10.1021/acs.jpca.2c02770)
Supplement: Supplementary file 1 — jp2c02770_si_001.pdf [file jp2c02770_si_001.pdf]

Supporting Information for

Matrix Isolation Study of Fumaric and Maleic Acids in Solid Nitrogen

Timur Nikitin,[1,*] Susy Lopes[1] and Rui Fausto[1,*]

[1]*CQC-IMS, Department of Chemistry, University of Coimbra, 3004-535 Coimbra, Portugal*

Index

Page

Table S1. Calculated (DFT(B3LYP)/6-311++G(d,p)) bond lengths (Å) and angles (º) for the conformers of maleic acid **S2**

Table S2. Calculated (DFT(B3LYP)/6-311++G(d,p)) bond lengths (Å) and angles (º) for the conformers of fumaric acid **S3**

Table S3. Calculated DFT(B3LYP)/6-311++G(d,p) anharmonic infrared spectra data for the conformers of maleic acid **S4**

Table S4a. Calculated DFT(B3LYP)/6-311++G(d,p) anharmonic infrared spectra data of conformers **I–V** of fumaric acid **S5**

Table S4b Calculated DFT(B3LYP)/6-311++G(d,p) anharmonic infrared spectra data of conformers **VI–X** of fumaric acid **S6**

Table S5. Experimental (N_2 and Ar matrix) and DFT(B3LYP)/6-311++G(d,p) calculated infrared data (anharmonic) for the *s-cis* conformer of acrylic acid **S7**

Figure S1. Fragment of near-infrared experimental spectrum of maleic acid in N_2 matrix, compared with the simulated anharmonic spectra for conformers **I** and **II** **S8**

Figure S2. Fragment of near-infrared experimental spectrum of fumaric acid in N_2 matrix, compared with the simulated anharmonic spectra for conformers **I–III** **S8**

Figure S3. Comparison of the results of irradiations of fumaric acid in N_2 matrix at 6909.5 and at 6936.5 cm^{-1} **S9**

Figure S4. Results of broadband UV irradiation of MA isolated in a N_2 matrix **S9**

Figure S5. Results of broadband UV irradiation of FA isolated in a N_2 matrix **S10**

* Corresponding authors: timur.nikitin@uc.pt (T.N.) // rfausto@ci.uc.pt (R.F.)

Table S1. Calculated (DFT(B3LYP)/6-311++G(d,p)) bond lengths (Å) and angles (º) for the conformers of maleic acid.[a]

	Conformers					
	**I**	**II**	**III**	**IV**	**VI**	**VII**
***Bond lengths* / Å**						
C_1=O_2	1.219	1.207	1.208	1.203	1.213	1.200
C_1–O_3	1.342	1.355	1.353	1.368	1.346	1.360
C_1–C_5	1.476	1.482	1.482	1.480	1.485	1.492
O_3–H_4	1.665	0.969	0.969	0.970	0.965	0.965
C_5–H_6	0.970	1.083	1.083	1.085	1.087	1.087
C_5=C_7	1.084	1.333	1.334	1.343	1.343	1.333
C_7–H_8	1.343	1.086	1.086	1.085	1.085	1.086
C_7–C_9	1.085	1.499	1.497	1.509	1.512	1.499
C_9=O_{10}	1.511	1.345	1.204	1.206	1.209	1.203
C_9–O_{11}	1.208	1.203	1.347	1.336	1.327	1.344
O_{11}–H_{12}	1.330	0.970	0.970	0.974	0.988	0.970
***Angles* / º**						
O_2=C_1–O_3	121.6	123.0	123.0	121.3	118.6	120.3
O_2–C_1–C_5	127.1	126.0	123.3	123.2	125.5	124.4
O_3–C_1–C_5	111.3	111.0	113.8	115.5	115.8	115.4
C_1–O_3–H_4	107.9	107.3	107.4	108.3	111.7	111.2
C_1–C_5–H_6	113.9	116.8	113.0	109.8	115.5	118.1
C_1–C_5=C_7	128.1	122.8	127.4	133.4	128.2	123.1
H_6–C_5=C_7	118.0	120.5	119.6	116.8	116.2	118.7
C_5=C_7–H_8	116.6	119.6	118.8	115.6	116.6	119.7
C_5=C_7–C_9	134.0	126.3	128.1	136.1	134.0	126.3
H_8–C_7–C_9	109.5	114.0	113.0	108.4	109.4	113.9
C_7–C_9=O_{10}	117.6	112.5	123.1	117.8	117.2	122.5
C_7–C_9–O_{11}	120.6	123.0	112.8	121.2	120.5	112.8
O_{10}=C_9–O_{11}	121.9	124.3	124.0	121.0	122.2	124.5
C_9–O_{11}–H_{12}	112.3	107.5	107.3	111.9	112.1	107.6
***Dihedrals* / º**						
O_2=C_1–C_3–H_4	0.0	0.0	-1.5	2.1	180.0	-177.2
C_5–C_1–O_3–H_4	180.0	179.6	179.4	-178.5	0.0	3.6
O_2=C_1–C_5–H_6	180.0	175.0	-10.4	6.8	180.0	-162.8
O_2=C_1–C_5=C_7	0.0	-4.1	169.4	-171.7	0.0	15.7
O_3–C_1–C_5–H_6	0.0	-4.6	168.8	-172.6	0.0	16.4
O_3–C_1–C_5–C_7	180.0	176.4	-11.5	9.0	180.0	-165.1
C_1–C_5–C_7–H_8	180.0	178.0	178.4	178.9	180.0	-177.0
C_1–C_5–C_7–C_9	0.0	2.1	2.1	0.5	0.0	-1.6
H_6–C_5=C_7–H_8	0.0	-1.0	-1.8	0.5	0.0	1.5
H_6–C_5=C_7–C_9	180.0	-176.9	-178.1	-177.8	180.0	176.9
C_5=C_7–C_9=O_{10}	180.0	-81.9	108.5	176.4	180.0	-111.5
C_5=C_7–C_9–O_{11}	0.0	102.8	-75.9	-3.3	0.0	72.9
H_8–C_7–C_9=O_{10}	0.0	101.9	-68.0	-2.0	0.0	64.1
H_8–C_7–C_9–O_{11}	180.0	-73.3	107.6	178.2	180.0	-111.4
C_7–C_9–O_{11}–H_{12}	0.0	179.7	-178.4	-1.0	0.0	-178.5
O_{10}=C_9–O_{11}–H_{12}	180.0	-5.1	-2.9	179.2	180.0	6.0

[a] See Figure 2 for atom numbering and pictures of the seven conformers of maleic acid. Form **V** does not exist at the DFT(B3LYP)/6-311++G(d,p) level.

Table S2. Calculated (DFT(B3LYP)/6-311++G(d,p)) bond lengths (Å) and angles (º) for the conformers of fumaric acid.[a]

	Conformers									
	**I**	**II**	**III**	**IV**	**V**	**VI**	**VII**	**VIII**	**IX**	**X**
Bond lengths /Å										
C_1–H_2	1.084	1.083	1.083	1.087	1.086	1.085	1.085	1.087	1.088	1.086
C_1=C_3	1.334	1.335	1.336	1.333	1.335	1.335	1.336	1.333	1.335	1.336
C_1–C_9	1.485	1.486	1.483	1.495	1.497	1.487	1.483	1.496	1.498	1.496
C_3–H_4	1.084	1.084	1.083	1.084	1.084	1.085	1.083	1.087	1.085	1.086
C_3–C_5	1.485	1.481	1.483	1.486	1.482	1.494	1.495	1.496	1.494	1.496
C_5=O_6	1.207	1.207	1.208	1.200	1.200	1.200	1.201	1.201	1.199	1.201
C_5–O_7	1.354	1.355	1.354	1.358	1.359	1.357	1.356	1.356	1.358	1.354
O_7–H_8	0.969	0.969	0.969	0.965	0.965	0.965	0.965	0.965	0.965	0.965
C_9=O_{10}	1.207	1.206	1.208	1.207	1.206	1.205	1.207	1.201	1.199	1.201
C_9–O_{11}	1.354	1.355	1.354	1.352	1.356	1.354	1.352	1.356	1.358	1.354
O_{11}–H_{12}	0.969	0.969	0.969	0.969	0.969	0.969	0.969	0.965	0.965	0.965
Angles / º										
H_2–C_1–C_3	121.1	121.9	121.7	119.1	120.1	123.0	122.8	119.0	121.1	122.0
H_2–C_1–C_9	118.2	117.6	114.4	119.9	119.4	116.3	112.8	120.3	118.5	112.7
C_3=C_1–O_9	120.8	120.5	124.0	120.9	120.5	120.7	124.3	120.7	120.4	125.2
C_1–C_3–H_4	121.1	120.9	121.7	120.9	120.6	119.9	120.8	119.0	119.6	122.0
C_1–C_3–C_5	120.8	124.2	124.0	120.6	124.2	124.9	124.8	120.7	124.5	125.2
H_4–C_3–C_5	118.2	114.9	114.4	118.5	115.2	114.9	114.2	120.3	115.5	112.7
C_3–C_5=O_6	125.9	123.5	123.4	124.3	124.3	121.8	121.6	124.0	121.9	121.2
C_3–C_5=O_7	110.9	113.5	113.6	115.4	115.4	117.5	117.7	115.5	117.2	117.8
O_6=C_5–O_7	123.2	122.9	123.0	120.4	120.4	120.7	120.7	120.5	120.9	121.0
C_5–O_7–H_8	107.4	107.2	107.2	111.3	111.3	110.8	111.0	111.3	110.7	111.2
C_1–C_9=O_{10}	125.9	126.0	123.4	125.6	123.6	125.8	123.2	124.0	123.9	121.2
C_1–C_9–O_{11}	110.9	110.9	113.6	111.0	113.4	110.7	113.5	115.5	115.6	117.8
O_{10}=C_9–O_{11}	123.2	123.2	123.0	123.3	122.9	123.4	123.3	120.5	120.5	121.0
C_9–O_{11}–H_{12}	107.4	107.4	107.2	107.6	107.2	107.6	107.4	111.3	111.6	111.2
Dihedrals / º										
H_2–C_1=C_3–H_4	180.0	180.0	180.0	180.0	180.0	-176.4	-176.5	180.0	176.3	180.0
H_2–C_1=C_3–C_5	0.0	0.0	0.0	0.0	0.0	-2.1	-2.0	0.0	2.7	4.4
C_9–C_1=C_3–H_4	0.0	0.0	0.0	0.0	0.0	1.8	2.1	0.0	-1.7	-4.4
C_9–C_1=C_3–C_5	180.0	180.0	180.0	180.0	180.0	176.1	176.6	180.0	-175.3	180.0
H_2–C_1–C_9=O_{10}	180.0	180.0	0.0	180.0	180.0	179.0	-2.3	180.0	174.7	26.6
H_2–C_1–C_9–O_{11}	0.0	0.0	180.0	0.0	0.0	-1.3	178.1	0.0	-4.8	-154.8
C_3=C_1–C_9=O_{10}	0.0	0.0	180.0	0.0	0.0	0.7	179.1	0.0	-7.3	-149.4
C_3=C_1–C_9–O_{11}	180.0	180.0	0.0	180.0	180.0	-179.6	-0.6	180.0	173.2	29.3
C_1=C_3–C_5=O_6	0.0	180.0	180.0	0.0	180.0	-147.8	-150.0	0.0	140.0	149.4
C_1=C_3–C_5–O_7	180.0	0.0	0.0	180.0	0.0	31.1	28.9	180.0	-38.5	-29.3
H_4–C_3–C_5=O_6	180.0	0.0	0.0	180.0	0.0	26.7	24.8	180.0	-33.8	-26.6
H_4–C_3–C_5–O_7	0.0	180.0	180.0	0.0	180.0	-154.4	-156.2	0.0	147.7	154.8
C_3–C_5–O_7–H_8	180.0	180.0	180.0	0.0	0.0	8.9	8.8	0.0	-10.9	-10.0
O_6=C_5–O_7–H_8	0.0	0.0	0.0	180.0	180.0	-172.2	-172.2	180.0	170.5	171.3
C_1–C_9–O_{11}–H_{12}	180.0	180.0	180.0	180.0	180.0	-180.0	179.9	0.0	-3.1	10.0
O_{10}=C_9–O_{11}–H_{12}	0.0	0.0	0.0	0.0	0.0	-0.3	0.2	180.0	177.4	-171.3

[a] See Figure 3 for atom numbering and pictures of the ten conformers of fumaric acid.

Table S3. Calculated DFT(B3LYP)/6-311++G(d,p) anharmonic infrared spectra data for the conformers of maleic acid.[a]

**I**		**II**		**III**		**IV**		**VI**		**VII**	
ν	I^{IR}	ν	I^{IR}	ν	I^{IR}	ν	I^{IR}	ν	I^{IR}	ν	I^{IR}
3564.6	107	3578.5	80	3587.1	88	3577.4	90	3620.8	64	3621.1	45
3130.9	548	3562.5	70	3559.1	73	3413.8	406	3075.6	537	3560.3	74
3058.6	2	3043.1	1	3075.3	1	3051.5	1	3064.3	3	3045.5	1
3004.2	<1	3009.6	<1	2990.9	<1	3064.5	<1	2965.2	6	2982.7	7
1758.6	164	1782.2	124	1778.6	451	1778.3	128	1768.6	216	1792.0	21
1710.5	254	1768.3	201	1763.1	806	1756.8	470	1727.0	187	1781.5	289
1643.8	31	1665.6	4	1654.7	33	1633.6	17	1626.3	52	1655.4	25
1418.7	63	1391.7	20	1390.9	38	1384.4	9	1383.5	150	1404.8	30
1377.3	134	1315.9	3	1329.9	155	1364.6	231	1420.3	16	1312.2	7
1308.2	22	1266.4	18	1301.3	21	1320.9	16	1288.1	1	1261.0	120
1275.9	3	1195.5	3	1211.2	12	1257.0	14	1257.4	545	1202.3	4
1194.4	26	1135.5	113	1140.1	478	1203.6	22	1199.8	27	1135.7	245
1143.2	371	1110.5	467	1126.8	904	1122.2	166	1129.3	4	1096.2	27
1071.9	1	991.7	2	996.0	7	1044.5	<1	1069.0	<1	967.6	1
925.8	16	945.0	6	917.0	54	875.0	12	929.2	12	939.7	25
888.4	42	817.5	30	825.0	201	872.0	59	901.4	72	823.3	15
866.7	9	820.5	22	812.4	75	788.4	2	849.9	<1	800.1	21
824.5	80	780.4	12	776.2	103	755.3	5	821.8	10	779.5	13
815.7	0	726.1	1	717.3	1	749.2	12	769.8	64	710.2	2
760.4	8	672.7	3	659.6	119	507.3	<1	760.7	4	672.8	9
671.0	31	588.7	95	543.3	593	575.0	6	610.9	4	540.9	81
604.2	19	551.1	38	516.9	484	655.6	7	591.0	1	555.3	26
585.1	29	542.8	138	588.4	358	565.8	68	585.0	4	519.5	4
527.7	68	459.6	3	483.2	129	310.1	251	486.3	99	454.3	14
388.1	1	435.1	30	432.7	266	395.7	4	386.4	6	314.1	71
300.1	9	284.5	1	278.9	19	264.5	27	290.3	<1	289.8	8
283.0	8	233.8	2	229.6	30	274.4	18	277.7	3	230.2	9
232.7	7	126.0	2	136.9	5	232.4	5	236.7	26	130.7	9
86.8	1	79.5	2	71.3	90	-95.2	21	78.0	4	83.6	4
38.2	3	24.8	1	11.8	764	22.3	9	18.3	4	15.0	1

[a] Form **V** does not exist at the DFT(B3LYP)/6-311++G(d,p) level.

Table S4a. Calculated DFT(B3LYP)/6-311++G(d,p) anharmonic infrared spectra data of conformers **I–V** of fumaric acid.

**I**		**II**		**III**		**IV**		**V**	
ν	I^{IR}	ν	I^{IR}	ν	I^{IR}	ν	I^{IR}	ν	I^{IR}
3582.4	0	3590.0	81	3582.2	0	3628.0	46	3623.5	45
3582.0	174	3582.0	90	3581.9	172	3581.4	88	3589.2	86
3078.9	0	3086.1	0	3090.1	1	3070.6	1	3073.2	1
3062.4	0	3065.2	0	3073.2	0	3036.4	2	3042.6	3
1765.8	0	1763.3	219	1749.2	0	1795.0	67	1787.5	74
1756.5	561	1745.5	218	1748.7	552	1756.8	328	1772.8	238
1671.1	0	1662.0	29	1652.4	0	1666.8	1	1656.8	29
1358.3	86	1322.9	92	1327.7	0	1308.2	408	1312.2	76
1326.4	0	1309.2	48	1306.6	90	1316.2	36	1320.9	21
1258.1	0	1277.7	20	1299.3	0	1253.3	180	1268.9	370
1216.0	49	1229.5	35	1252.7	27	1213.1	0	1229.1	7
1127.9	0	1144.9	109	1156.1	0	1121.4	245	1144.9	200
1104.4	652	1113.4	264	1114.5	416	1099.1	149	1097.2	44
1001.7	45	1004.9	46	1005.7	47	992.9	47	1000.2	46
951.7	0	928.5	20	915.7	0	946.2	15	921.6	44
920.5	0	916.5	1	890.5	0	911.6	1	909.0	2
895.1	4	881.8	5	887.4	16	891.3	14	878.0	4
782.9	54	781.1	52	780.2	25	773.4	34	773.3	31
681.4	0	676.7	3	648.5	0	698.6	11	675.1	1
666.9	0	659.4	10	644.2	0	679.8	10	661.4	2
616.9	219	600.2	95	600.2	63	606.2	28	596.8	48
599.7	82	588.4	60	572.8	229	546.8	5	553.3	99
557.5	0	546.9	18	547.8	0	538.3	71	551.9	11
540.4	43	544.5	91	536.1	23	477.4	115	455.1	47
368.9	0	384.9	3	405.0	0	368.8	12	386.2	8
267.1	0	258.3	1	260.4	0	269.8	1	264.1	3
148.7	0	165.5	0	138.9	0	147.2	6	152.0	4
142.5	0	131.2	0	136.3	3	137.8	7	134.0	4
129.6	3	116.8	0	127.1	0	127.7	10	128.0	3
53.7	4	42.0	0	41.6	3	54.2	4	49.4	0

Table S4b. Calculated DFT(B3LYP)/6-311++G(d,p) anharmonic infrared spectra data of conformers **VI–X** of fumaric acid.

**VI**		**VII**		**VIII**		**IX**		**X**	
ν	I^{IR}	ν	I^{IR}	ν	I^{IR}	ν	I^{IR}	ν	I^{IR}
3625.1	33	3615.0	32	3808.6	0	3812.2	56	3804.6	86
3580.3	91	3584.7	92	3808.4	122	3806.2	41	3804.4	0
3063.5	0	3076.2	0	3158.9	2	3186.9	4	3170.6	2
3039.5	3	3043.3	4	3155.6	0	3132.7	7	3165	0
1787.6	162	1778.5	66	1826.6	0	1830.8	227	1823.1	0
1769.2	219	1753.1	262	1816.0	517	1828.1	332	1820.1	656
1651.6	18	1648.0	1	1704.9	0	1686.5	37	1680.5	0
1328.7	43	1349.8	17	1349.7	0	1350.2	68	1326.9	0
1276.2	174	1296.3	108	1324.7	861	1305.8	393	1319.4	0
1271.3	123	1267.2	109	1297.2	0	1291.5	502	1305.5	630
1223.3	6	1252.8	43	1261.5	331	1259.1	63	1275.6	244
1150.9	6	1159.0	41	1141.6	0	1175.5	20	1190	0
1109.5	312	1118.9	168	1127.5	70	1124.5	32	1164.8	32
997.8	38	996.7	27	992.2	54	1007.9	50	1010.4	68
937.7	14	908.9	7	956.9	0	946.1	24	929.7	0
900.7	2	892.4	7	910.4	0	900.1	5	897.7	52
873.9	24	877.7	26	909.2	42	885.7	28	892.4	0
767.3	26	769.0	16	772.4	12	766.7	16	768	19
681.1	18	677.3	10	699.6	0	695	8	702.7	0
665.3	5	638.0	7	640.4	0	656.3	4	615.2	25
596.4	52	606.1	28	624.1	23	607.3	16	603.3	0
576.3	39	560.2	49	558.1	5	520.5	14	548.4	12
539.3	80	514.1	85	444.3	229	487.2	108	483.9	198
426.8	38	439.5	28	423.0	0	426.6	62	476.4	0
381.7	36	391.7	32	372.1	0	396.8	35	394.5	0
253.4	2	247.7	2	277.4	0	261.1	5	255	0
158.9	4	143.1	7	147.5	28	142.8	24	153.4	23
114.9	8	156.5	13	128.2	24	128.6	13	123.8	0
126.4	6	99.8	1	81.0	0	117.6	6	116.4	24
40.4	2	39.9	10	38.4	12	39.7	3	48	17

Table S5. Experimental (N_2 and Ar matrix) and DFT(B3LYP)/6-311++G(d,p) calculated infrared data (anharmonic) for the *s-cis* conformer of acrylic acid, with proposed assignments.[a]

	Experimental		Calculated	
Approximate description	N_2 matrix	Ar matrix[60]	ν	I^{IR}
ν(OH)	3600.0/3588.0	3566.7/3564.2/3561.3	3576.5	70.3
$\nu(CH_2)$as	n.obs.	n.obs.	3105.6	1.3
ν(CH)	n.obs.	n.obs.	3033.3	3.6
$\nu(CH_2)$s	n.obs.	n.obs.	2991.3	2.4
ν(C=O)	1779.0	1764.1/1762.5/1746.0	1764.5	249.5
$\nu(C{=}C)+\delta(CH_2)$	1652.0	1663.0/1635.0/1633.9	1661.8	19.6
$\delta(CH_2)$	1396.0	1411.6/1410.7/1408.2	1405.1	66.8
δ(COH)+ν(CO)	n.obs	1323.4	1316.7	11.9
$\delta(CH)+w(CH_2)$	n.obs.	1252.0	1262.7	0.7
δ(COH)	1122.0	1194.0/1190.4/1132.2/ 1121.1	1119.9	346.1
$w(CH_2)$	999.0/996.0	1060.7	1060.7	37.5
τ(C=C)	n.obs.	994.0/986.9	1000.1	29.2
$\gamma(CH_2)$	n.obs.	973.4/9671.4	999.1	23.9
ν(CC)	827.0	830.2/828.7	821.5	7.4
γ(C=O)	n.obs.	812.4	818.6	36.8
τ(CO)	616.0	620.5/616.3	621.6	13.1
δ(O=CO)	611.0	610.1/607.0	613.3	79.4
δ(CC=O)	n.i.	491.8	486.7	19.4
γ(C=O)+γ(CH)	n.i.	448.8	471.1	27.5
δ(C=CC)+δ(CC=O)	n.i.	n.i.	276.1	2.0
τ(CC)	n.i.	n.i.	114.3	1.2

[a] Wavenumbers (cm^{-1}), calculated intensities (km mol^{-1}), s = symmetric; a = antisymmetric; ν = stretching; δ = in-plane bending; γ = out-of-plane bending; τ = torsion; n.obs. = not observed; n.i. = not investigated.

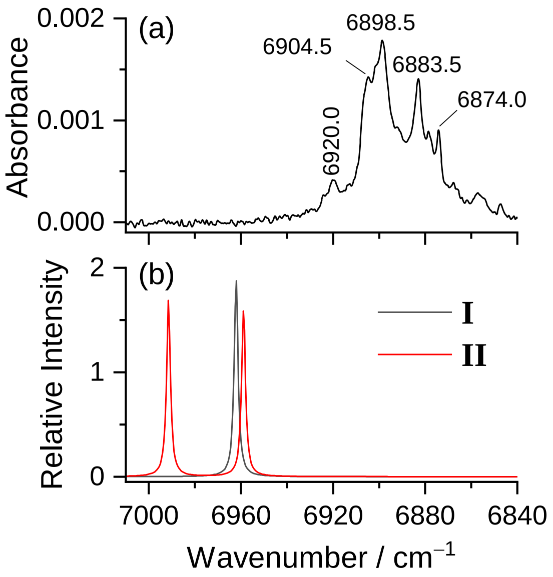

Figure S1. Fragment of near-infrared experimental spectrum of maleic acid isolated in a nitrogen matrix (a) compared with the simulated anharmonic spectra for conformers **I** and **II** (b) obtained at the DFT(B3LYP)/6-311++G(d,p) level.

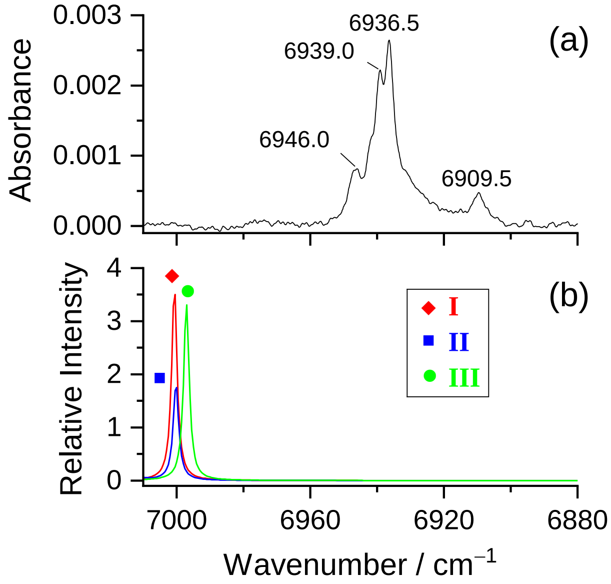

Figure S2. Fragment of near-infrared experimental spectrum of fumaric acid isolated in a nitrogen matrix (a) compared with the simulated anharmonic spectra for conformers **I–III** (b) obtained at the DFT(B3LYP)/6-311++G(d,p) level.

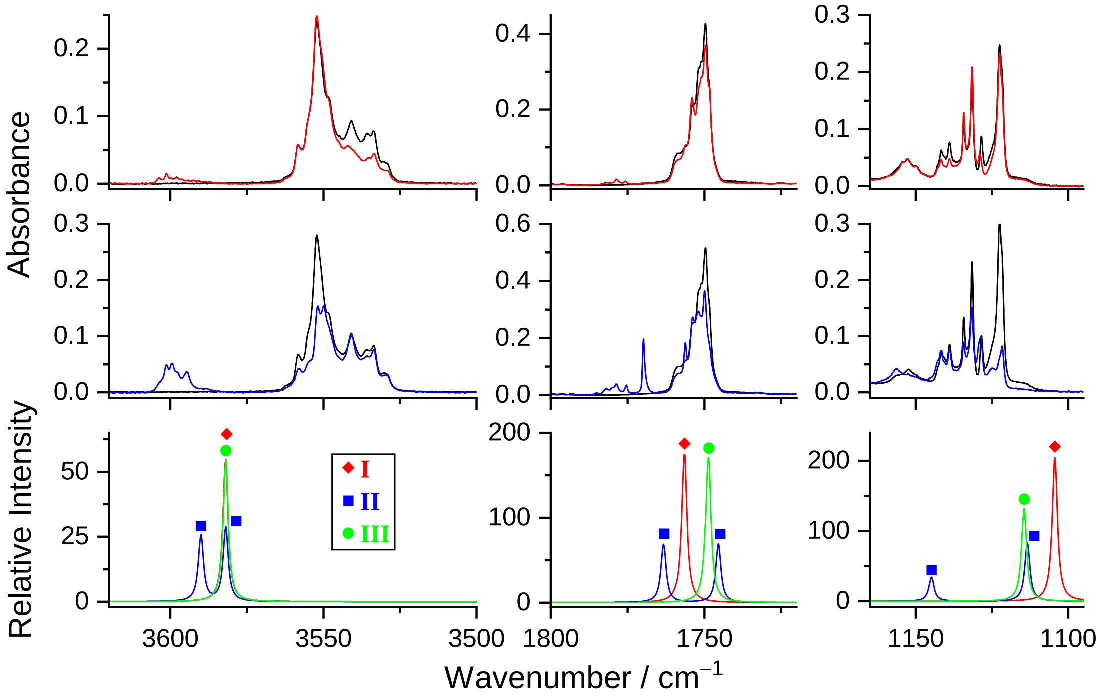

Figure S3. Experimental FTIR spectrum of FA isolated in N_2 matrix at 15 K after deposition (black) and after irradiation at 6909.5 cm^{-1} (red) (a) and after irradiation at 6936.5 (blue) (b); theoretical infrared spectra with the anharmonic frequencies of conformers **I** (red), **II** (blue), and **III** (green) (c) calculated at the DFT(B3LYP)/6-311++G(d,p) level.

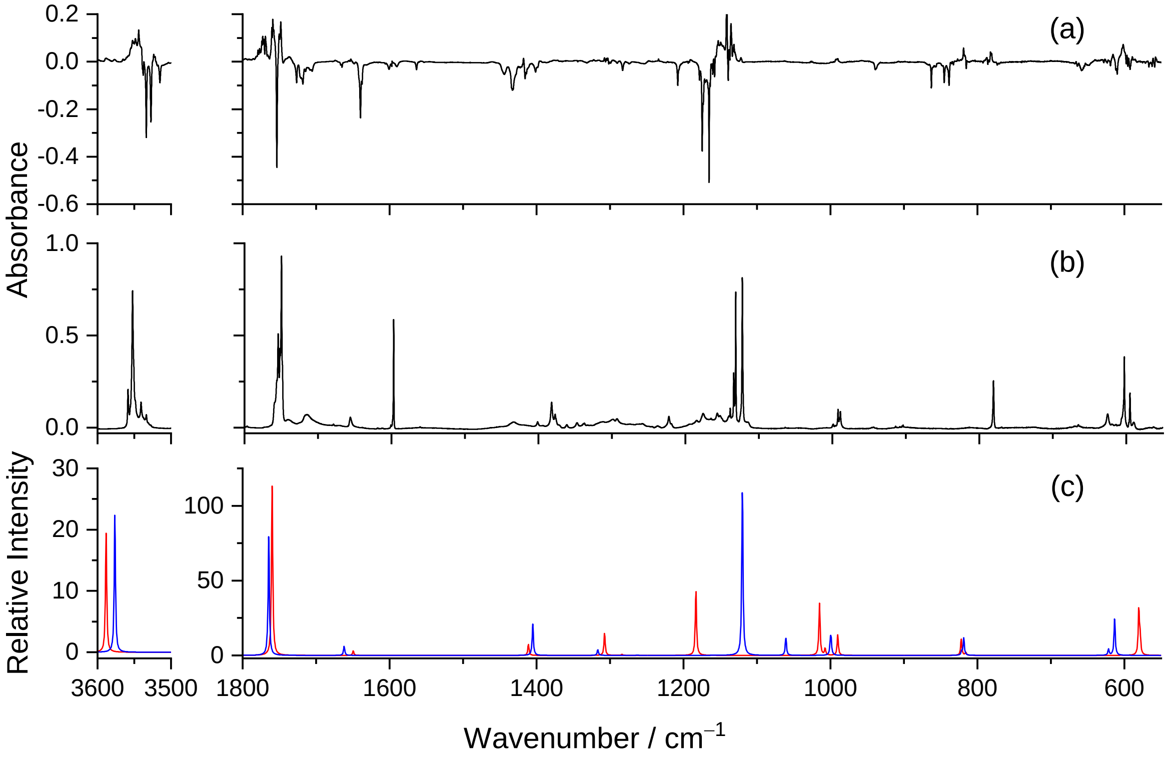

Figure S4. Experimental FT-IR spectrum of MA showing the effects resulting from broadband irradiation (λ > 235 nm) (a), experimental FT-IR spectrum of FA isolated in a N_2 matrix at 7.5 K after deposition (b), and simulated anharmonic IR spectra of *s-cis* (red) and *s-trans* (blue) acrylic acid computed at the DFT(B3LYP)/6-311++G(d,p) level of theory (c).

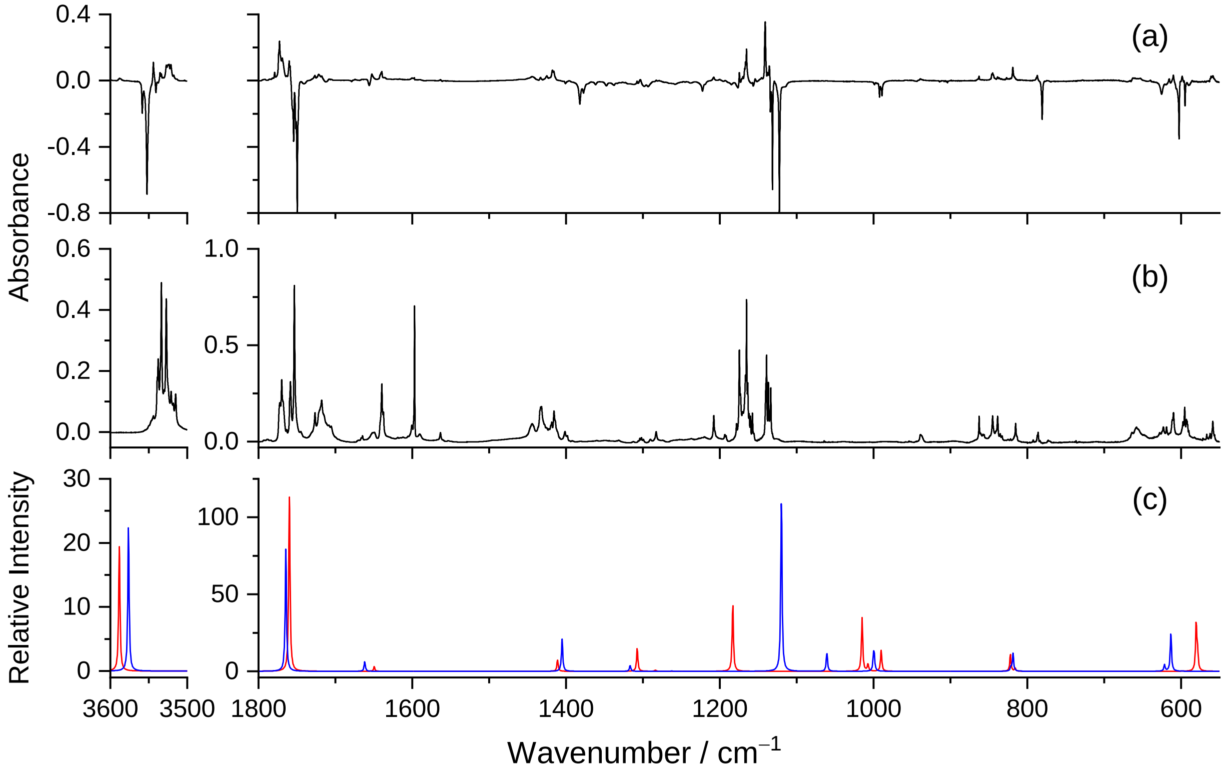

Figure S5. Experimental FT-IR spectrum of FA showing the effects resulting from broadband irradiation (λ > 235 nm) (a), experimental FT-IR spectrum of MA isolated in a N_2 matrix at 7.5 K after deposition (b), and simulated anharmonic IR spectra of *s-cis* (red) and *s-trans* (blue) acrylic acid computed at the DFT(B3LYP)/6-311++G(d,p) level of theory (c).
